# Abnormalities in cortical pattern of coherence in migraine detected using ultra high-density EEG

**DOI:** 10.1093/braincomms/fcab061

**Published:** 2021-04-02

**Authors:** Alireza Chamanzar, Sarah M Haigh, Pulkit Grover, Marlene Behrmann

**Affiliations:** 1 Department of Electrical and Computer Engineering, Carnegie Mellon University, Pittsburgh, PA, USA; 2 Neuroscience Institute, Carnegie Mellon University, Pittsburgh, PA 15213, USA; 3 Department of Psychology, University of Nevada, Reno, NV 89557, USA; 4 Institute for Neuroscience, University of Nevada, Reno, NV 89557, USA; 5 Department of Psychology, Carnegie Mellon University, Pittsburgh, PA, USA

**Keywords:** cortical coherence, high-density electroencephalography, migraine, resting state, sensory evoked potentials

## Abstract

Individuals with migraine generally experience photophobia and/or phonophobia during and between migraine attacks. Many different mechanisms have been postulated to explain these migraine phenomena including abnormal patterns of connectivity across the cortex. The results, however, remain contradictory and there is no clear consensus on the nature of the cortical abnormalities in migraine. Here, we uncover alterations in cortical patterns of coherence (connectivity) in interictal migraineurs during the presentation of visual and auditory stimuli and during rest. We used a high-density EEG system, with 128 customized electrode locations, to compare inter- and intra-hemispheric coherence in the interictal period from 17 individuals with migraine (12 female) and 18 age- and gender-matched healthy control subjects. During presentations of visual (vertical grating pattern) and auditory (modulated tone) stimulation which varied in temporal frequency (4 and 6 Hz), and during rest, participants performed a colour detection task at fixation. Analyses included characterizing the inter- and intra-hemisphere coherence between the scalp EEG channels over 2-s time intervals and over different frequency bands at different spatial distances and spatial clusters. Pearson’s correlation coefficients were estimated at zero-lag. Repeated measures analyses-of-variance revealed that, relative to controls, migraineurs exhibited significantly (i) faster colour detection performance, (ii) lower spatial coherence of alpha-band activity, for both inter- and intra-hemisphere connections, and (iii) the reduced coherence occurred predominantly in frontal clusters during both sensory conditions, regardless of the stimulation frequency, as well as during the resting-state. The abnormal patterns of EEG coherence in interictal migraineurs during visual and auditory stimuli, as well as at rest (eyes open), may be associated with the cortical hyper-responsivity that is characteristic of abnormal sensory processing in migraineurs.

## Teaser

High-density EEG signals revealed abnormal brain connectivity in interictal migraine sufferers versus controls. This atypical pattern was noted during auditory and visual stimulation, and during rest. The results may aid diagnosis and treatment.

## Graphical Abstract

**Graphical Abstract:**
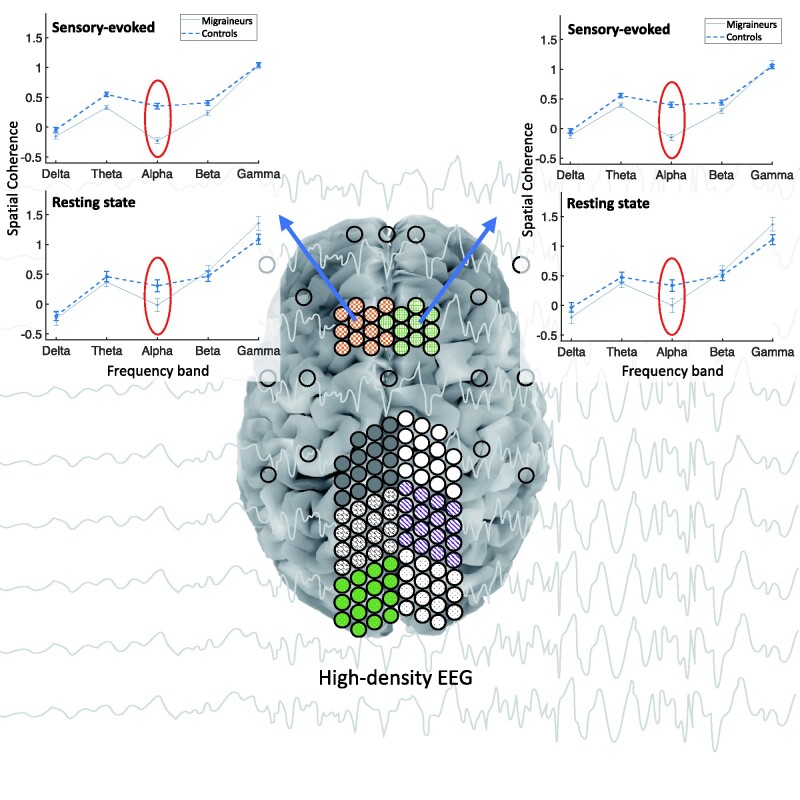

## Introduction

More than 38 million people in the USA suffer from migraine,^1^ a neurovascular condition that manifests as episodes of headache, accompanied by other autonomic and neurological symptoms. The incidence of migraine worldwide is high, with current estimates of ∼10% of people affected.^2^ Neurophysiological, morphometric and functional imaging studies have focussed on uncovering the pathogenesis, biomarkers and effective treatments for this sometimes debilitating disorder. Notwithstanding this large scientific effort, there is still no consensus on the underlying mechanism/s that give rise to migraines.

### Connectivity

One candidate mechanism for migraine is altered functional cortical connectivity. Many studies of functional connectivity have been conducted using resting-state fMRI^3–8^ or EEG recordings,^9^^,^^10^ for a review, see De Tommaso.^11^ A key result has been the demonstration of connectivity alterations in multiple cortical regions in the pre-ictal and ictal migraine phases,^12^ suggesting that abnormal functional connectivity is not a consequence of the migraine attack itself and may even potentially be causal. Although findings from resting recordings in migraine show inconsistencies across studies,^13^ among the more consistent findings are abnormal connectivity between brain areas associated with pain, and between sensory areas,^14^ with strength of connectivity related to headache severity (for a review, see Schwedt et al.^15^) The altered functional connectivity,^16^ particularly the stimulus-evoked connectivity, as reflected in greater cortical hyper-responsiveness^17–19^ may be associated with migraine based on altered sensory processing as in photophobia and phonophobia^96^.

The evidence for sensory hyper-responsivity in migraineurs is based on heightened neural responses to sensory stimuli compared with controls.^20–22^ Hyper-responsiveness has been observed in both fMRI^23–26^ and EEG responses,^27^^,^^28^ including evidence of reduced habituation to repeated stimuli which suggests heightened sensitivity to sensory input.^29^ Much of the available evidence for cortical hyper-responsiveness in migraine is gleaned from studies using stimuli such as checkerboards, repetitive flashes or pattern reversal stimulation.^17^^,^^18^^,^^20^^,^^30^^,^^31^ For example, one study documented larger amplitude steady-state visual evoked potentials in interictal migraineurs than controls, and these stimuli harmonized oscillations of different cortical areas, including visual areas.^32^ The bias towards abnormal visual system activity may be related to the prevalence of auras which are more likely to be visual.^33^

On the other hand, few studies have investigated the neural correlates of auditory processing in migraine. One study reported that children with migraine exhibited deficits in tests of acoustic timing,^34^ while another found that otoacoustic emissions were lower in women with migraine, but only at lower temporal frequencies.^35^ These findings may be related to the abnormal electrophysiological responses to auditory stimuli. Auditory brainstem responses and auditory evoked potentials, which correspond with the reduced grey matter in the brainstem,^36^ were also abnormal in migraine.^37^^,^^38^ Together, these observations indicate abnormalities in auditory and visual processing in migraine that may be associated with or impacted by the phonophobia and photophobia of migraineurs.

### Frequency-specific differences

Of particular relevance for the present study, some EEG studies have reported differences in specific frequency bands in migraineurs compared with controls, but this may depend on where on the scalp the abnormalities appear, and whether the responses were recorded at rest or were stimulus-evoked. For example, relative to controls, reports suggest an increase in the power of delta or theta band both during rest,^39–41^ and during visual evoked responses in interictal migraineurs.^42^^,^^43^ Other investigations have noted low overall coherence in theta band,^44^ low inter-hemispheric coherence in delta, beta and alpha bands, and high intra-hemispheric coherence (in all frequency bands) in female interictal migraineurs during rest (using low-density EEG^45^) In addition, decreased power in the alpha band^40^^,^^43^ and greater asymmetry in alpha band power have also been reported during resting recordings,^46^^,^^47^ and during steady-state visual evoked potentials stimulation.^41^^,^^48^^,^^49^ In another study, using high frequency flash stimuli, increased phase synchronization in alpha band in interictal migraine patients without aura was reported.^50^ Decreased cortical coherence after photic stimulation, and increased coherence during the resting state in female interictal migraine with aura have also been reported in all of the frequency bands except gamma.^51^ Last, a more recent study has reported lower EEG power and coherence in fronto-central and parietal regions in interictal migraine patients, during resting state, and in all of the frequency bands except gamma.^9^ There are many potential explanations for these contradictory results including alterations in the method of data acquisition, e.g. in differences between rest and sensory-evoked recordings.

### The current study

The goal of the current investigation was to compare cortical connectivity during sensory-evoked and rest recordings in individuals with migraine versus headache-free controls, to investigate the extent to which abnormal connectivity is evident in migraine. Owing to the heightened sensory sensitivity in migraine, abnormal functional connectivity may, in fact, be more evident during sensory stimulation compared with rest. We chose to use EEG because its superior temporal resolution permits us to assess connectivity in different frequency bands. We acquired data using a custom-designed EEG cap with ultra-high-density coverage (∼14 mm centre-to-centre electrode distance) over visual, parietal and frontal regions.^28^^,^^52^ EEG signals were obtained from participants under three conditions: visual stimulation (vertical grating patterns), auditory stimulation (modulated tone) and resting state.

Although EEG amplitude measurements and calculation of power spectra are simple to compute, we elected to study coherence, which is considered to be a reliable measure of synchronization of the electro-cortical activities^53^ and which has been used to investigate connectivity in migraine previously.^9^^,^^44^^,^^45^^,^^51^ Because spatial coherence drops as a function of distance between electrodes, we analysed the estimated coherence as a function of inter-electrode distance (*i.d.* or link length). We chose two commonly used stimulation frequencies of 4 and 6 Hz,^54–57^ for both visual and auditory stimulation to determine whether any differences across sensory modalities or between groups were temporally frequency-specific. If sensory stimulation primarily drives abnormal connectivity in migraine, then group differences would be evident during evoked but not rest conditions. If, however, the disturbed connectivity occurs across-the-board and independent of input, then we might see coherence differences in both the two evoked and the rest recordings.

## Materials and methods

### Participants

Seventeen adults with migraine (mean age 27.6 years old; range 19–54 years; 12 female), and 18 age- and gender-matched headache-free controls (mean age 27.9 years old; range 19–54 years) were recruited from Carnegie Mellon University and from the surrounding Pittsburgh area for the study. Control participants were only included if, by self-report, they were headache-free and either had never had a headache or had infrequent headaches that were less than moderately painful and had no co-occurring sensory disturbances.

All individuals with migraine satisfied the International Headache Society criteria with 12 participants classified as having migraine with aura (third edition of international classification of headache disorders 1.2) and 5 as having migraine without aura (third edition of international classification of headache disorders 1.1). Three individuals in the migraine-with-aura group were medicated (two took Triptans and the other had Botox). We excluded these patients from all the analyses in this paper to ensure that the medication could not affect the results (migraine severity and frequency information is shown in Table 1). In addition, in our analyses, we pooled together migraineurs, with and without aura. The small sample of migraineurs without aura (*n* = 5) makes the comparisons between migraineurs with and without aura underpowered. Abnormal patterns of coherence should still be detectable with a group of 14 participants with migraine (mean age 25.9 years old; range 19–47 years; 9 female) given that, in Mendonça-de-Souza et al.^51^ data from 11 migraine patients were successfully used for a spatial coherence analysis. No participant had a neurological or psychological diagnosis (except for migraine), no previous severe head injury or concussion, had normal hearing, and normal or corrected-to-normal vision by self-report. All procedures were approved by the Carnegie Mellon University Institutional Review Board. Written informed consent form was received from each subject before starting each recording session. Participants were paid $50 for their participation.

**Table 1:** Severity and frequency of migraines in the 14 individuals with migraine who were not on any migraine medication

	Mean	SD
Duration of migraine (h)	10.82	17.95
Pain severity (out of 10)	7.14	1.18
Days since last migraine	59.92	84.55
Approximate number of migraines per year	31.87	35.22
When did they start (years)	8.23	8.06
MIDAS (days; 0–5 means little or no disability)	6.64	6.12

MIDAS, migraine disability assessment.

### Stimuli

MATLAB^98^ and the Psychtoolbox extension^58–60^ were used to generate and present the stimuli.

#### Visual stimulation

**Figure 1:**
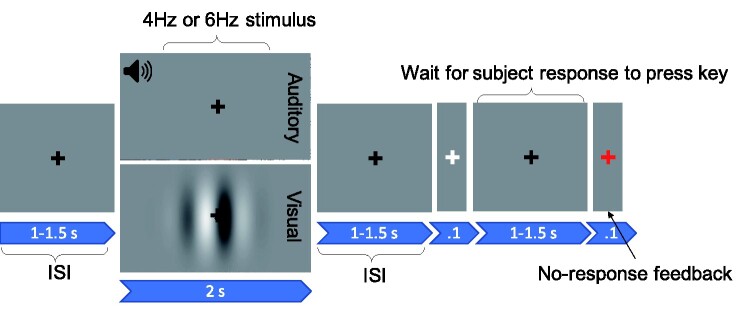
**Structure of auditory and visual trials.** A fixation cross appeared centrally, jittered for between 1 and 1.5 s. A stimulus, either auditory or visual, was then presented for 2 s followed by a 1–1.5 s inter-stimulus-interval (ISI), consisting of a grey screen and a fixation cross. Participants pressed the spacebar whenever the cross flashed white (for 0.1 s). If they did not respond, the fixation cross turned red (for 0.1 s).

Vertical sinusoidal-wave achromatic gratings were presented at 0.05 cpd, subtending 5.7 degrees of visual angle in diameter in the centre of the screen at a viewing distance of 1 m (see Fig. 1). Gratings were filtered using a spatial 2D Gaussian filter. The gratings alternated contrast at 4 Hz or at 6 Hz for 2 s followed by an inter-stimulus interval that varied between 1 and 1.5 s. Each temporal frequency was presented 100 times, and the order of temporal frequency was randomized. A central fixation cross (0.5 cpd) was presented throughout the duration of the experiment and was superimposed on the central grating.

#### Auditory stimuli

The auditory stimuli were 1 kHz tones, modulated by a sinusoidal 4 or 6 Hz carrier frequency and were presented for 2 s. The stimuli were sampled at 44.1 kHz with 16-bit resolution. Each modulator frequency was presented 100 times. Tones were separated by an inter-stimulus interval between 1 and 1.5 s where no sound was played. Tones were presented over insert earphones (Etymotic Research, Inc.). A grey screen with a black central fixation cross was presented for the duration of the experiment (see Fig. 1).

#### Resting state

The resting state condition consisted of a single black fixation cross shown in the centre of the grey screen for the duration of the recording.

### Procedure

The resting EEG recording was always completed prior to the stimulation conditions so that the sensory stimuli would not ‘contaminate’ the resting EEG signal. During the resting recording, participants kept their eyes open and fixated on the black central cross. Six blocks of 2-min (12 min total) were recorded, with a self-paced break in between each block.

The order of the visual and auditory recordings was counterbalanced across participants. For both recordings, as shown in Fig. 1, stimuli were presented for 2 s and preceded and followed by a grey screen with a fixation cross (inter-stimulus interval of 1–1.5 s; random with uniform distribution). Participants were asked to ignore the stimuli and to attend to the fixation cross. They were required to press the spacebar whenever the cross flashed white (for 0.1 s), which occurred randomly on 10% of trials. If the participant did not respond, the fixation cross turned red for 0.1 s. Four blocks of 50 trials (25 of the 4 Hz stimuli, 25 of the 6 Hz) were presented and, in each block, the stimulation frequencies were randomly ordered. Each block of trials took ∼3 min, with the result that each participant contributed ∼6 min of EEG data for each of the auditory and visual conditions.

### EEG recording

**Figure 2:**
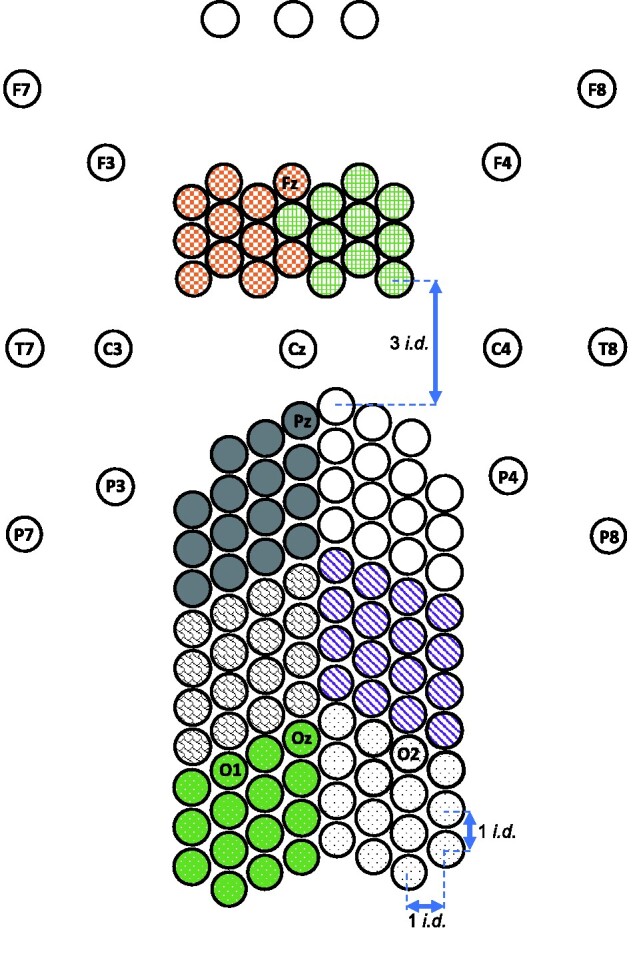
**A 2D map of electrode locations.** 10-20 electrode labels are included for reference. Coloured patterns indicate clusters used for analysis: left and right frontal, parietal, occipito-parietal, and occipital areas. Scale adjusted for illustration. Contiguous electrodes have equal vertical and horizontal inter-electrode distances (i.d.) in this 2D map.

A 128 channel BioSemi ActiveTwo system (BioSemi, Amsterdam, the Netherlands) was used to record the EEG signals at 512 Hz sampling frequency using a 24-bit A/D converter. We used a custom-designed high-density EEG nylon cap with electrodes specifically positioned so as to cover central occipital, parietal and frontal areas (the locations of alterations typically reported in previous studies of migraine) with high resolution. Further details of the electrode locations are available in our previous paper.^28^ Electrodes were placed ∼1.4 cm distance from one another. A 2D map of the electrode locations is shown in Fig. 2. An additional seven electrodes were placed around the head: to detect the electrooculography signals, four electrodes were placed around the eyes: one electrode above and one below the right eye, and one on the outer canthi of each eye. For recording of electrocardiography signals, one electrode was placed on the collar bone. Two electrodes were placed on the mastoids. Standard BioSemi common mode sense and driven right leg electrodes were used as online references for all electrodes.

To take advantage of the high-density EEG montage, for the analysis, the EEG electrodes were grouped into eight spatial clusters. These are shown in Fig. 2 as right and left occipital (dotted pattern), occipito-parietal (shingle pattern and stripes), parietal (solid grey and white) and frontal electrodes (checkerboard and grid pattern) (for successful use of these clusters, see Haigh et al.^28^).

### Response time

The reaction times (RT) to the colour change at fixation were analysed first. Trials with RTs longer than 1 s or with no-response were excluded (∼15% of total trials), including ∼12% of the trials for migraineurs and ∼16% of trials for control participants (this proportion of trials is statistically independent of the group type, i.e. migraineurs and controls: *P*  > 0.1, chi-square test; Pearson^61^). Trials with RTs that preceded the colour change were also excluded (∼0.2% of the trials), which likely were a result of anticipatory behaviour. The remaining RTs were averaged for each participant separately for each modality (visual and auditory) and each stimulation frequency (4 and 6 Hz).

### EEG preprocessing

EEG data were preprocessed using the EEGLAB^62^ and ERPLAB^63^ toolboxes in MATLAB^98^: (i) EEG data were re-referenced offline to the average of the two mastoid electrodes and a zero-phase Butterworth filter was used to filter the signals between 0.1 and 100 Hz; (ii) noisy channels were detected visually and interpolated (this was done in 0.95% of electrodes from the migraine group and 0.3% from the control group); (iii) independent component analysis was used to identify and remove eye-related artefacts (blinks and horizontal eye movements), and heartbeat; (iv) for the visual and auditory trials, the first 2 s after the stimulus onset were extracted, and, for the resting state trials, 2-s non-overlapping time intervals were extracted; and (v) these 2-s time intervals were passed through zero-phase Kaiser bandpass filters to extract 5 frequency bands of delta (0.5 − 3 Hz), theta (4 − 7 Hz), alpha (8 − 12 Hz), beta (12 − 30 Hz) and gamma (30 − 100 Hz). A lowpass filter with cut-off frequency of 30 Hz is standardly used for noisy EEG data^64^. However, we chose to keep the high-frequency gamma band given that: (a) the Biosemi ActiveTwo system has active electrodes and it can tolerate high electrode impedances^65^, (b) the participants were seated inside a Faraday cage during the EEG recording to reduce the electromagnetic interferences^66^ and (c) we detected and interpolated noisy channels, as stated in Step (ii) of the preprocessing.

### Coherence

We estimated the Pearson’s correlation coefficients (PCCs) at zero-lag (*n* = 0) as a measure of coherence between electrical activities of each pair of electrodes (X and Y) for each frequency band (f). PCCs take values in the range of [−1, 1]:

(1) $$\rho_{XY}^{f}\left(m,n=0\right)=\frac{E\left[\left(\begin{array}{c} \begin{array}{c} X_{f}\left(m\right)-{\mu_{X}}_{f}\left(m\right) \end{array} \end{array}\right){\left(\begin{array}{c} Y_{f}\left(m+n\right)-{\mu_{Y}}_{f}\left(m+n\right) \end{array}\right)}^{*}\right]}{{\sigma_{X}}_{f}\left(m\right){\sigma_{Y}}_{f}(m+n)}$$

where μ and σ are the mean and standard deviation of *X* and *Y*, which are estimated using *N* repeated trials, i.e. *N* bandpass filtered 2-s time intervals were used as sample functions of these two stochastic processes *X* and *Y*:

(2) $${\hat{\rho }}_{XY}^{f}\left(m\right)=\frac{1}{\left(N-1\right){{\hat{\sigma }}_{X}}_{f}\left(m\right){{\hat{\sigma }}_{Y}}_{f}\left(m\right)}\sum_{i=1}^{N}\left(X_{f}^{i}\left(m\right)-{\hat{\mu }}_{X_{f}}\left(m\right)\right)\left(\begin{array}{c} Y_{f}^{i}\left(m\right)-{\hat{\mu }}_{Y_{f}}\left(m\right) \end{array}\right)$$

where we have used unbiased estimators of variances, covariances, and mean of these random processes:

(3) $${{\hat{\mu }}_{X}}_{f}\left(m\right)=\frac{1}{N}\sum_{i=1}^{N}X_{f}^{i}(m),$$

(4) $${{\hat{\mu }}_{Y}}_{f}\left(m\right)=\frac{1}{N}\sum_{i=1}^{N}Y_{f}^{i}(m),$$

(5) $${{{\hat{\sigma }}^{2}}_{X}}_{f}\left(m\right)=\frac{1}{N-1}\sum_{i=1}^{N}{\left(\begin{array}{c} X_{f}^{i}\left(m\right)-{{\hat{\mu }}_{X}}_{f}\left(m\right) \end{array}\right)}^{2},$$

(6) $${{{\hat{\sigma }}^{2}}_{Y}}_{f}\left(m\right)=\frac{1}{N-1}\sum_{i=1}^{N}{\left(\begin{array}{c} Y_{f}^{i}\left(m\right)-{{\hat{\mu }}_{Y}}_{f}\left(m\right) \end{array}\right)}^{2},$$

the estimated PCCs in (1) are functions of time (m) and frequency (f). We side-stepped the unrealistic stationarity assumption for EEG signals. We consider the absolute value of PCCs in our analyses, since both correlation (positive sign) and anti-correlation (negative sign) capture the coherence of activities for each pair of EEG electrodes.

#### Spatial analysis

We averaged the absolute value of estimated PCCs over 2-s time intervals to obtain a spatial map of coherence. PCCs were grouped based on the length of their corresponding links in the 2D map of electrodes (see Fig. 2). In this 2D map, the vertical and horizontal inter-electrode distances of each pair of contiguous electrodes are equal (except the gaps between frontal and parietal clusters). This inter-electrode distance (i.d., see Fig. 2) is used as a unit of distance for calculation of ‘link lengths’ in this study, i.e. link length 1 (21–40 $i.d.^{2}$), link length 2 (41–60 $i.d.^{2}$), link length 3 (61–80 $i.d.^{2}$) and link length 4 (≥81 $i.d.^{2}$), where $i.d.^{2}$ is the unit of squared Euclidean distance. We excluded the links with the smallest length (≤20) from the analysis to remove the spurious high correlations in nearby electrodes.^67^ For each cluster of electrodes (see Fig. 2), we defined two measures: (i) inter-hemispheric, defined as the PCCs calculated between electrodes of a cluster and the electrodes placed on the other hemisphere, averaged for each link length, and (ii) intra-hemispheric, defined as the PCCs calculated between electrodes of a cluster and the electrodes placed on the same hemisphere, averaged for each link length.

### Statistical analysis

To ascertain the effect of modality and/or stimulation frequency on the detection RT in migraine patients, a three-way mixed-model ANOVA was used with two levels of modality (auditory and visual) and stimulation frequency (4 and 6 Hz) as within-subject factors and group (migraineur and control subject) as the between-subject variable, and averaged RT as the dependent variable (see Response time section for more details).

For measures of EEG coherence, we used two separate six-way mixed-model ANOVAs for each of the stimulation frequencies (4 and 6 Hz) with frequency bands (delta, theta, alpha, beta and gamma), link lengths (21–40, 41–60, 61–80 and ≥81), two levels of modalities (visual and auditory), eight spatial clusters (left and right occipital, occipito-parietal, parietal and frontal electrodes) and hemisphere (intra/inter) as within-subject factors, and group (migraineurs and control subjects) as the between-subject factor. Also, a five-way mixed-model ANOVA was conducted on the rest dataset with four within-subject factors (EEG frequency bands, link lengths, spatial clusters, hemisphere), and group as a between-subjects factor. We performed *post hoc* power analysis for these ANOVA tests and, as evident, our sample size is large enough to reveal significant interactions with high statistical power (see Supplementary Note C for details).

We checked the normality (using Kolmogorov−Smirnov test with α=0.05; Massey^68^) and homoscedasticity (using Shapiro-Wilk test with α=0.05; Shapiro and Wilk^69^) assumptions for mixed-model ANOVA tests used in this study and noted that the distribution of estimated spatial coherence was leptokurtically skewed. The violation of these assumptions has been corrected using the hyperbolic transformation (HP transformation) defined in Tsai et al.,^70^ closely following the steps in this paper to estimate the parameters of HP transformation based on maximum likelihood estimation and the transformed data are used in the coherence analysis. HP transformation was not applied on the RTs in paper, since they were normally distributed (*P* > 0.5) and were homoscedastic (*P* > 0.2). Least significant difference (LSD) *post hoc* test was used to explore any significant interactions in the mixed-model ANOVAs. MATLAB^98^ and Unixstat^71^ were used for all analyses in this study.

### Data availability

The anonymized raw EEG dataset of the participants in this research are made available online on KiltHub, Carnegie Mellon University’s online data repository^95^ (DOI: 10.1184/R1/12636731).

## Results

We first assessed the RTs and then spatio-temporal pattern of hemispheric coherence in the evoked tasks. This was followed by a similar analysis of the resting-state EEG data. In this section, we use ‘*M*’ and ‘*C*’ to report the results for migraine patients and controls, respectively.

### Response time

Each participant’s RTs were averaged over trials separately for stimulation frequency (4 and 6 Hz) and modality (visual and auditory), and these factors were subjected to an ANOVA with group (migraineur and control) as the between-subject variable. Migraineurs had significantly faster response times (mean = 449 ms) compared with the headache-free control subjects [mean = 527 ms; main effect of group: *F*(1,31) = 13.57, *P* < 0.001]. There were no significant interactions of the group with any other factor. The same group difference was observed when comparing just RTs of migraineurs with aura (mean = 451 ms) against their matched headache-free controls [mean = 587 ms; main effect of group: *F*(1,17) = 22.41, *P* < 0.0001].

### Coherence

#### Measures of coherence during visual and auditory stimulation

##### Omnibus interactions of 4 Hz stimulation frequency

The mixed-model ANOVA for 4 Hz stimulation frequency revealed a five-way interaction of group×link lengths×spatial clusters×hemisphere×modalities [*F*(21,546) = 1.67, *P* < 0.04], a four-way interaction of group×hemisphere×frequency bands×link lengths [*F*(12,312) = 1.91, *P* < 0.04] and a four-way interaction of group×hemisphere×frequency bands×spatial clusters [*F*(28,728) = 1.68, *P* < 0.02], along with several lower level interactions which are all subsets of the aforementioned higher-level interactions.

##### Omnibus interactions of 6 Hz stimulation frequency

The mixed-model ANOVA for 6 Hz stimulation frequency revealed similar interactions: a four-way interaction of group×hemisphere×frequency bands×link lengths [*F*(12,312) = 2.85, *P* < 0.001], a four-way interaction of group×hemisphere×frequency bands×spatial clusters [*F*(28,728) = 1.78, *P* < 0.01] and a three-way interaction of group×link lengths×spatial clusters [*F*(21,546) = 1.71, *P* < 0.03], along with several lower level interactions which are all subsets of the aforementioned higher-level interactions. To characterize the interactions, multiple *post hoc* comparisons were conducted using the LSD test.

#### Coherence differences in different frequency bands and link lengths

**Figure 3:**
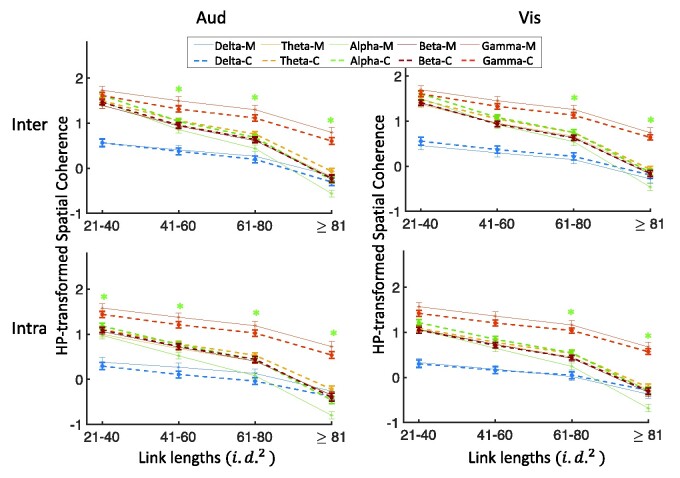
**Four-way coherence interaction of group×hemisphere×frequency bands×link lengths for 4 Hz stimulation frequency.** Comparison of spatial coherence between individuals with migraine and controls for the visual and auditory stimulation frequency of 4 Hz as a function of link lengths for each of the five frequency bands, each of the hemisphere (inter/intra) and groups. HP-transformed spatial coherence is shown using dashed lines for controls and solid lines for migraineurs. Asterisks show the significant group differences for each link length (on the *x*-axis) and each frequency band (colours of asterisks are matched with the frequency bands), based on LSD (*P* < 0.05) *post hoc* test (*M*, migraineurs; *C*, controls).

In this part, we explored and compared the effects of different link lengths and frequency bands on the spatial coherence in migraineurs against controls. Figure 3 shows the spatial coherence values for stimulation frequency of 4 Hz (visual and auditory separately) as a function of link lengths for each of the five frequency bands, hemisphere (intra/inter) and groups (the four-way interaction of group×hemisphere×frequency bands×link lengths). In comparison with controls, migraineurs showed significantly lower spatial coherence for visual stimulation frequency of 4 Hz in the alpha frequency band for long inter-/intra-hemisphere connections of ≥61 $i.d.^{2}$(*P* < 0.04; *M* = −0.088 ± 0.046, *C* = 0.215 ± 0.040), and lower spatial coherence for auditory stimulation frequency of 4 Hz in the alpha frequency band for medium and long inter-/intra-hemisphere connections of ≥41 $i.d.^{2}$ (*P* < 0.05; *M* = 0.089 ± 0.039, *C* = 0.368 ± 0.033) and for short intra-hemisphere connections of 21–40 $i.d.^{2}$ (*P* < 0.04; *M* = 0.975 ± 0.076, *C* = 1.184 ± 0.059).

Supplementary Figure 1 shows the results of the same analysis spatial coherence values for stimulation frequency of 6 Hz (visual and auditory separately) as a function of link lengths for each of the five frequency bands, hemisphere (intra/inter) and groups (the four-way interaction of group×hemisphere×frequency bands×link lengths). Similar to the observed trends in 4 Hz stimulation frequency, in comparison with controls, migraineurs showed significantly lower spatial coherence for visual stimulation frequency of 6 Hz in the alpha frequency band for long inter-/intra-hemisphere connections of ≥61 $i.d.^{2}$(*P* < 0.02; *M* = −0.156 ± 0.046, *C* = 0.193 ± 0.039), for medium-length intra-hemisphere connections of 41–60 $i.d.^{2}\;$(*P* < 0.02; *M* = 0.589 ± 0.071, *C* = 0.825 ± 0.058), and for auditory stimulation frequency of 6 Hz in the alpha frequency band for long inter-/intra-hemisphere connections of ≥81 $i.d.^{2}\;$(*P* < 0.01; *M* = −0.668 ± 0.058, *C* = 0.371 ± 0.055) and for medium-length intra-hemisphere connections of 61–80 $i.d.^{2}\;$(*P* < 0.01; *M* = 0.136 ± 0.073, *C* = 0.422± 0.063).

To summarize, migraineurs showed significantly lower spatial coherence in the alpha frequency band for long inter-electrode distances during the visual and auditory stimuli (for both 4 and 6 Hz stimulation frequencies).

#### Coherence differences in different link lengths and spatial clusters

**Figure 4:**
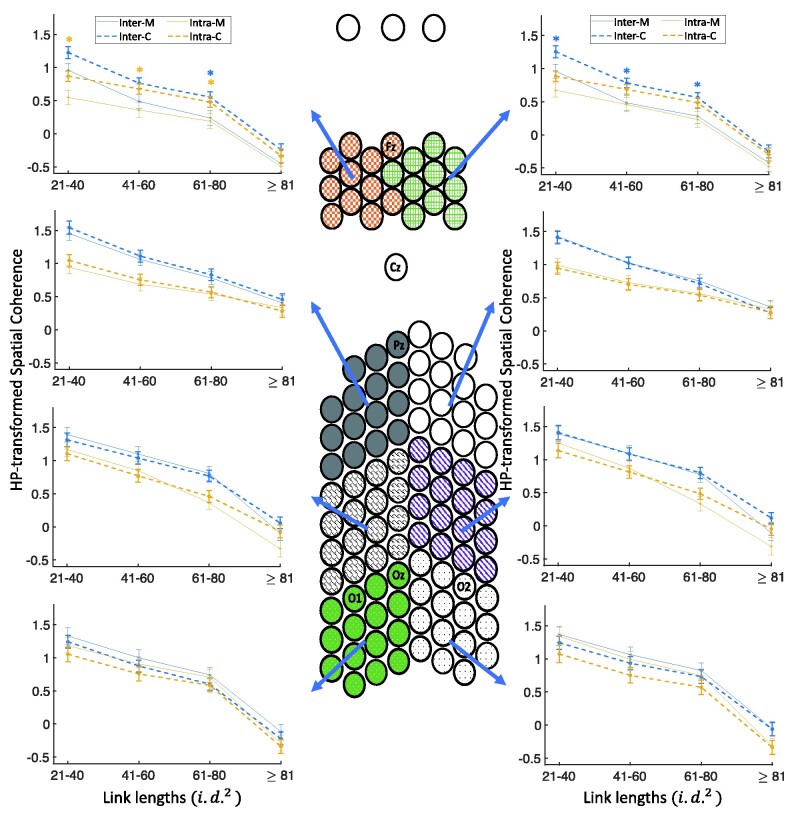
**Five-way coherence interaction of group×link lengths×spatial clusters×hemisphere×modalities for 4 Hz stimulation frequency.** Comparison of spatial coherence between migraineurs and controls for the visual stimulation frequency of 4 Hz as a function of link lengths for each of the eight spatial clusters, hemisphere (intra/inter) and groups (Fig. 5 shows the results for the auditory stimulation). HP-transformed spatial coherence is shown using dashed lines for controls and solid lines for migraineurs. Asterisks show the significant group differences for each link length (on the *x*-axis) and each hemisphere (colours of asterisks are matched with the inter- and intra-hemisphere), based on LSD (*P* < 0.05) *post hoc* test (*M*, migraineurs; *C*, controls).

**Figure 5:**
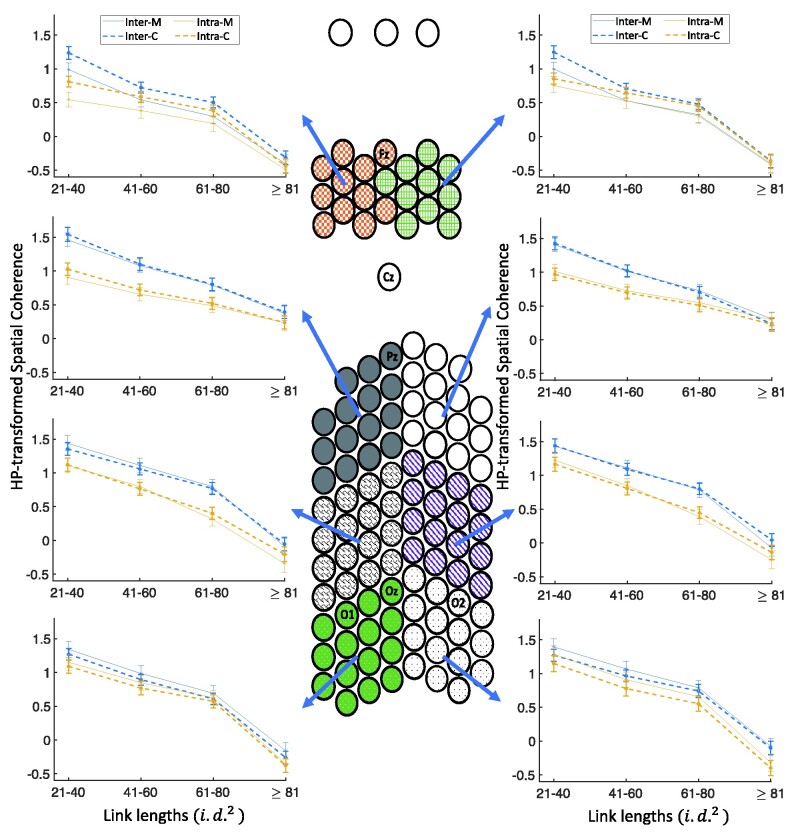
**Five-way coherence interaction of group×link lengths×spatial clusters×hemisphere×modalities for 4 Hz stimulation frequency.** Comparison of spatial coherence between migraineurs and controls for the auditory stimulation frequency of 4 Hz as a function of link lengths for each of the eight spatial clusters, hemisphere (intra/inter), and groups (Fig. 4 shows the results for the visual stimulation). HP-transformed spatial coherence is shown using dashed lines for controls and solid lines for migraineurs. Asterisks show the significant group differences for each link length (on the *x*-axis) and each hemisphere (colours of asterisks are matched with the inter- and intra-hemisphere), based on LSD (*P* < 0.05) *post hoc* test (*M*, migraineurs; *C*, controls).

To find out which cluster or clusters show significant group differences in long inter-electrode distances, as observed in the previous part, we explored the coherence differences for each spatial cluster at each link length. Figure 4 shows the spatial coherence values for visual stimulation frequency of 4 Hz as a function of link lengths for each of the eight spatial clusters, hemisphere (intra/inter) and groups (the five-way interaction of group×link lengths×spatial clusters×hemisphere×modalities). Figure 5 shows this interaction for auditory stimulation frequency of 4 Hz. In comparison with controls, migraineurs showed significantly lower spatial coherence for visual stimulation frequency of 4 Hz in both frontal clusters for long inter-hemisphere connections of 61–80 $i.d.^{2}\;$(*P* < 0.05; *M* = 0.260 ± 0.079, *C* = 0.558 ± 0.055), in the right frontal cluster for short- and medium-length inter-hemisphere connections of 21–60 $i.d.^{2}\;$(*P* < 0.05; *M* = 0.723 ± 0.079, *C* = 1.020 ± 0.062), and in the left frontal cluster for short and long intra-hemisphere connections of 21–80 $i.d.^{2}\;$(*P* < 0.05; *M* = 0.366 ± 0.065, *C* = 0.676 ± 0.047).

Supplementary Figure 2 shows the results of similar analysis for visual and auditory stimulation frequency of 6 Hz. Similar to the results for 4 Hz stimulation frequency, using the LSD test, in comparison with controls, migraineurs showed significantly lower spatial coherence for visual stimulation frequency of 6 Hz in both frontal clusters for medium-length and long connections of 21–80 $i.d.^{2}\;$(*P* < 0.04; *M* = 0.505 ± 0.033, *C* = 0.755 ± 0.025), and in the right occipito-parietal cluster for long links of ≥81 $i.d.^{2}$ (*P* < 0.02; *M* = −0.204 ± 0.092, *C* = 0.058 ± 0.062). For auditory stimulation frequency of 6 Hz, migraineurs showed significantly lower spatial coherence only in the left frontal clusters for short links of 21–40 $i.d.^{2}$(*P* < 0.03; *M* = 0.788 ± 0.075, *C* = 1.026 ± 0.067).

To summarize, independent of stimulation frequency, migraineurs showed significantly lower spatial coherence predominantly in frontal clusters for long inter-electrode distances during the visual stimulation (the trend is in the same direction for auditory stimulation).

#### Coherence differences in different frequency bands and spatial clusters

**Figure 6:**
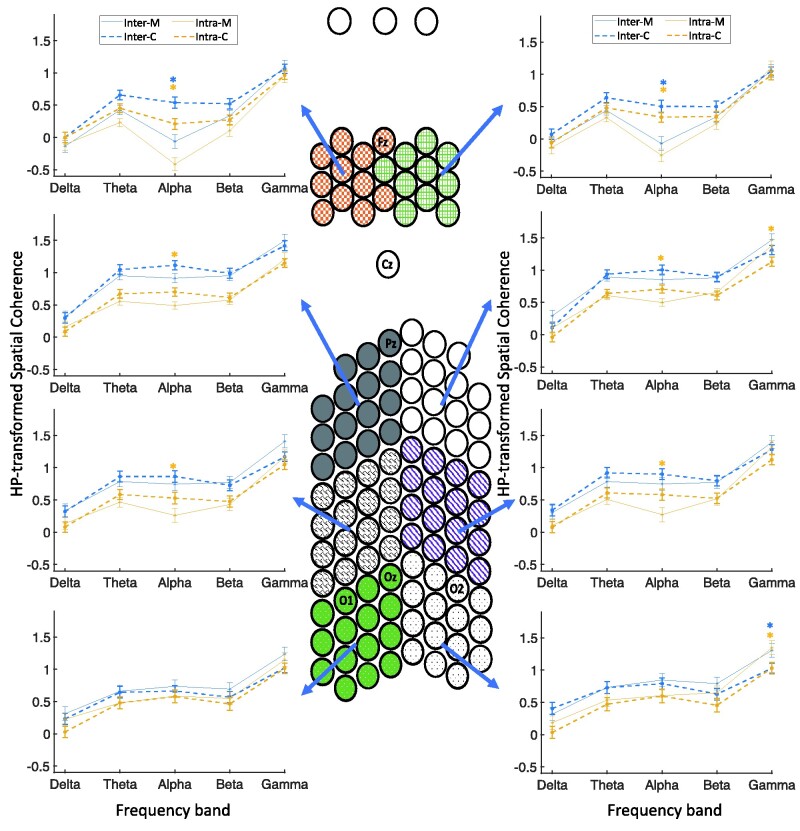
**Four-way coherence interaction of group×hemisphere×frequency bands×spatial clusters for 4 Hz stimulation frequency.** Comparison of spatial coherence between individuals with migraine and controls for the stimulation frequency of 4 Hz as a function of frequency bands for each of the eight spatial clusters, hemisphere (intra/inter) and groups. HP-transformed spatial coherence is shown using dashed lines for controls and solid lines for migraineurs. Asterisks show the significant group differences for each frequency band (on the *x*-axis) and each hemisphere (colours of asterisks are matched with the inter- and intra-hemisphere), based on LSD (*P* < 0.05) *post hoc* test (*M*, migraineurs; *C*, controls).

The primary focus here is on whether the coherence results differ as a function of frequency band and hemisphere and if so, given the observed significant effect of frontal electrodes in the previous part, whether this also differs by spatial clusters. Figure 6 shows the spatial coherence values for stimulation frequency of 4 Hz as a function of frequency bands for each of the eight spatial clusters, hemisphere (intra/inter) and groups (the four-way interaction of group×hemisphere×frequency bands×spatial clusters). Note that the data are collapsed across modality as this factor is not included in this interaction. Based on the LSD results for 4 Hz stimulation frequency, compared with controls, migraineurs showed significantly lower spatial coherence in the frontal clusters for the alpha frequency band for both inter- and intra-hemisphere connections (*P* < $10^{-5}$; *M* = −0.200 ± 0.051, *C* = 0.398 ± 0.044), and in the parietal and occipito-parietal clusters for the alpha frequency band for intra-hemisphere connections (*P* < 0.05; *M* = 0.381 ± 0.044, *C* = 0.630 ± 0.039). In addition, migraineurs showed significantly higher spatial coherence in the right parietal and occipital clusters for the gamma frequency band for intra-hemisphere connections (*P* < 0.03; *M* = 1.364 ± 0.070, *C* = 1.080 ± 0.057), and in the right occipital cluster for the gamma frequency band for inter-hemisphere connections (*P* < 0.04; *M* = 1.305 ± 0.107, *C* = 1.028 ± 0.077).

Supplementary Figure 3 shows the results of the same analysis for stimulation frequency of 6 Hz, again collapsed across modality. Similar to the results for 4 Hz stimulation frequency, in comparison with controls, migraineurs showed significantly lower spatial coherence in the frontal clusters for the alpha frequency band for both inter- and intra-hemisphere connections (*P* < 0.0001; *M* = −0.180 ± 0.050, *C* = 0.354 ± 0.044), in the parietal and occipito-parietal clusters for the alpha frequency band for intra-hemisphere connections (*P* < 0.04; *M* = 0.361 ± 0.045, *C* = 0.622 ± 0.040), and in the left frontal cluster for the theta frequency band for inter-hemisphere connections (*P* < 0.05; *M* = 0.425 ± 0.076, *C* = 0.664 ± 0.076). In addition, migraineurs showed significantly higher spatial coherence in the right parietal and occipital clusters for the gamma frequency band for intra-hemisphere connections (*P* < 0.04; *M* = 1.381 ± 0.070, *C* = 1.117 ± 0.056), in the right occipital cluster for the gamma frequency band for inter-hemisphere connections (*P* < 0.04; *M* = 1.326 ± 0.106, *C* = 1.052 ± 0.075), in the left occipital cluster for the delta frequency band for intra-hemisphere connections (*P* < 0.03; *M* = 0.215 ± 0.087, *C* = −0.084 ± 0.087) and in the right parietal cluster for the delta frequency band for inter-hemisphere connections (*P* < 0.03; *M* = 0.253 ± 0.082, *C* = 0.025 ± 0.070). In other words, migraineurs showed significant lower inter- and intra-hemisphere coherence in the frontal clusters for the alpha frequency band, and lower intra-hemisphere coherence in the parietal and occipito-parietal clusters for the alpha frequency band, regardless of the modality and stimulation frequency.

Taken together, these findings revealed that during the evoked condition largely for both modalities and stimulation frequencies, in comparison with control participants, migraineurs showed lower spatial coherence usually in both inter- and intra-hemispheres, that was more evident in frontal clusters and for longer links, and to a greater extent in the alpha frequency range.

### Measures of coherence during resting-state recordings

#### Omnibus interactions of resting-state

To assess whether the group differences in coherence measures were a product of sensory stimulation, we conducted the same analyses with four within-subject factors (EEG frequency bands, link lengths, spatial clusters, hemisphere), and group as a between-subjects factor on the resting-state EEG data. There was a four-way interaction of group×frequency bands×spatial clusters×link length [*F*(84,2184) = 1.69, *P* < 0.001] along with several lower level interactions which are all subsets of the aforementioned higher level interaction.

#### Coherence differences in different frequency bands and link lengths across spatial clusters

**Figure 7:**
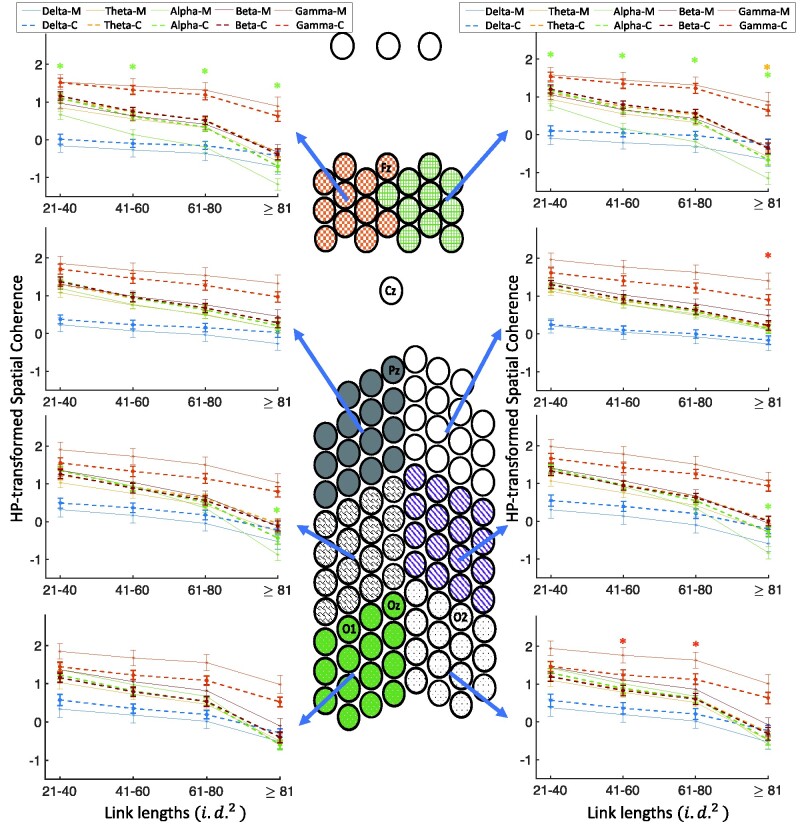
**Four-way resting-state coherence interaction of group×frequency bands×spatial clusters×link lengths.** Comparison of spatial coherence between individuals with migraine and controls during the resting-state recording as a function of link lengths for each of the five frequency bands, each of the eight spatial clusters and groups. HP-transformed spatial coherence is shown using dashed lines for controls and solid lines for the migraine patients. Asterisks show the significant group differences for each link length (on the *x*-axis) and each frequency band (colours of asterisks are matched with the frequency bands), based on LSD (*P* < 0.05) *post hoc* test (*M*, migraineurs; *C*, controls).

Similar to the coherence analyses in the previous part, we explored the effects of link length and frequency band on the spatial coherence differences across different electrode clusters (see Fig. 7). In comparison with controls, migraineurs showed significantly lower spatial coherence in both frontal clusters for the alpha frequency band for all link lengths of ≥21 $i.d.^{2}$(*P* < 0.03; *M* = −0.123 ± 0.067, *C* = 0.364 ± 0.059), and in the right frontal cluster for the theta frequency band for the longest connections (*P* < 0.04; *M* = −0.586 ± 0.097, *C* = −0.334 ± 0.101). In addition, in both occipito-parietal clusters, migraineurs showed significantly lower coherence in the alpha frequency band for the longest connections (*P* < 0.02; *M* = −0.858 ± 0.113, *C* = −0.361 ± 0.103), significant higher coherence in the gamma frequency band for the longest connections in the right parietal cluster (*P* < 0.04; *M* = 1.403 ± 0.211, *C* = 0.898 ± 0.138), and significant higher coherence in the gamma frequency band for medium-length connections (41–80 $i.d.^{2}$) in the right occipital cluster (*P* < 0.05; *M* = 1.700 ± 0.145, *C* = 1.184 ± 0.098).

In summary, investigation of pairwise differences in coherence measures (with correction) revealed mainly consistent differences between groups in rest and in sensory-evoked recordings, i.e. the frontal clusters in migraineurs showed significant lower spatial coherence in the alpha frequency band. The results of similar comparison of the migraineurs with aura against their controls largely replicated the results reported above (see Supplementary Note A for details).

### Coherence normalization

One aspect of the analysis that warrants further investigation concerns the fact that absolute values of PCCs have a different range of values for different inter-electrode distances (very small values for long links and *vice versa*). To ensure that this does not affect the results of the coherence analysis, we repeated the entire spatial coherence analysis using the normalized PCCs. Since the correlations drop exponentially as the inter-electrode distance increases (see Supplementary Fig. 11), we used an exponential regression to fit an exponential function ($ae^{bx}+c$) to the absolute value of PCCs which are averaged for each inter-electrode distance. Then PCCs are normalized by this exponential function of inter-electrode distance.

The results of the normalized coherence analysis are included in the Supplementary Note B, which are remarkably similar to those of the unnormalized results reported in this section. This confirms that the different ranges of spatial coherence values for different inter-electrode distances (small values for long links and vice versa), do not affect the reported results in this study.

## Discussion

The goal of this investigation was to evaluate comprehensively the cortical dynamics in individuals with migraine compared with headache-free controls during rest and during sensory stimulation. We examined spatial coherence (connectivity) at each of the EEG frequency bands using signals acquired from a customized ultra-high-density EEG system. Responses were measured to visual and to auditory stimulation, and at rest (with eyes-open). Signals, both normalized and unnormalized, were compared within and between hemispheres as a function of two stimulation frequencies (4 and 6 Hz) as well as distance between electrodes. Participants completed a colour change detection at fixation in the visual and auditory stimulation trials to ensure that attention was controlled. Several major results emerged.

Migraineurs were significantly faster at responding to the fixation cross compared with controls. This may be related to the cortex being hyper-responsive in migraine, as seen in increased visual evoked EEG to checkerboards, repetitive flashes or pattern reversal stimulation.^17^^,^^18^^,^^20^^,^^30^^,^^31^

Interestingly, the differences in spatial coherence networks between migraineurs and headache-free controls were evident in both the sensory-evoked recordings and the resting-state recordings. Other studies have previously reported abnormal functional connectivity in migraine during rest.^9^^,^^44^^,^^45^^,^^51^ The abnormal connectivity in sensory-evoked recordings was consistent with the heightened sensory sensitivities (photophobia and phonophobia) that are characteristic of migraine even in the interictal period,^23^^,^^26^^,^^28^^,^^35^^,^^72–76^ and, indeed, the spatial coherence networks were similar across visual and auditory-evoked recordings, suggesting altered cortical dynamics across modalities in migraine.^27^^,^^77^ We note the subtle differences in the topography of the functional connectivity between visual and auditory stimulation, and further exploration of differences across sensory modalities in migraine may help to elucidate similarities and differences across modalities in migraine.

Surprisingly, there has been rather minimal investigation on auditory functioning in migraine, and findings of functional dynamics in resting and visual-evoked recordings are rather inconsistent.^44^^,^^45^^,^^50^ Here, we examined auditory and visual evoked signals across the five EEG frequency bands. The key results from the spatial coherence analysis, a measure of synchronization of the electro-cortical activities,^53^ revealed that migraineurs showed significantly *lower* spatial coherence of alpha-band neural activities during both the auditory and visual stimuli, as well as the resting-state recordings, in comparison with the controls. This profile was especially evident in a wide range of distances (short and long connections) between the frontal clusters of scalp electrodes and other clusters (for both inter- and intra-hemisphere connections). Desynchronization of connections (lower coherence) in the alpha band, suggests greater functional activity,^78–80^ and is consistent with the cortex being hyper-responsive in migraine.^81^

A possible account of desynchronization in migraine may be that of thalamocortical dysrhythmia: underactivity in thalamic nuclei that results in reduced neural synchrony across the brain,^82^ especially in low-frequency oscillations (theta range). Thalamocortical dysrhythmia has been linked to migraine as a potential cause for the cortical hyper-responsiveness and sensory disruptions.^16^^,^^77^^,^^83^ However, the desynchronization in alpha band signals in migraine appears to be in contradiction with reported increased phase synchronization in alpha band in patients with interictal migraine and without aura.^50^ It is possible that those who experience migraine without aura exhibit different patterns of activity compared with those with aura. The majority of our migraine participants (9 out of 14) experience auras, and so there may be an impact of aura that generates different neural signatures in the alpha response. Our current migraine sample is too small to assess the specific effect of aura on coherence, but this may be an interesting avenue for future study.

Identifying atypical electrophysiology in individuals with migraine is useful from both a basic science and a translational perspective. Uncovering the alterations in cortical dynamics can ascertain which neural signatures are related to different disorders. Spatial coherence metrics such as those identified here have the potential to diagnose migraineurs. For example, lower connectivity in the theta band was successfully used to predict group membership using a classifier.^44^ This reduction in connectivity is consistent with the findings we have obtained. Changes in spectral power may also be able to predict migraine onset and self-testing at home with a portable, relatively inexpensive EEG system is becoming feasible.^84^ Further exploration into how connectivity changes over the course of the migraine cycle will help with identification of when the next migraine attack will occur allowing for more targeted prophylactic treatment.^85^

In addition, identifying the cortical dynamics that are unique to migraine will help ascertain the mechanisms that contribute to the migraine pathogenesis. These mechanisms could then lead to targeted treatments to reduce the severity of, or perhaps even prevent, migraine.^86^^,^^92^ Using interventions that can disrupt migraine-related network activity and decrease heightened cortical responsivity, such as transcranial direct current stimulation,^88^ can potentially offer non-pharmacological prophylactic treatments. Migraine can also occur or become appreciably worse after trauma (termed post-traumatic headache), particularly after a mild traumatic brain injury or concussion.^89^ Identifying the mechanisms that contribute to migraine can help identify the changes in the brain post-trauma, potentially leading to improved treatment.

This study has several limitations. First, as mentioned earlier, our study includes a small number of migraine patients without aura. Hence, we pooled the participants (and also compared the migraineurs with aura against their controls; see Supplementary Note A) and did not compare these two groups separately, but further comparison of these groups is clearly warranted. Second, while having high-density of scalp electrodes in patches over occipital, parietal and frontal areas was appropriate for assessing visual and auditory stimulation, some spatial sensitivity may have been lost for the rest recording. Due to time constraints during the experimental session, we were limited to 128 channels, and so chose to focus on the areas of the scalp most related to visual and auditory processing. For further analysis of coherence equally across the scalp, an EEG cap with high-density electrodes with equally spaced coverage across the head is required. Third, the PCCs used in this study as the measure of coherence do not capture the direction of correlation and information flow (unlike other measures such as Granger causality^87^). Accounting for the direction of coherence will require a larger sample size. In addition, measures of coherence do not explain the relative importance of amplitude and phase covariance, while a phase-locking value can measure the phase synchronization in neural activities.^64^ Last, we were unable to control the time since the last migraine attack, the time to the next attack or the number of years over which the individual suffered from migraines. Previous studies have shown that the general time course of migraine attacks normalizes neural functioning during the attack and that the abnormalities increase with longer time since attack.^32^^,^^83^^,^^89–97^ Assessing changes in spatial coherence over the migraine cycle and longitudinally may highlight the predictive power of these network changes preceding a migraine attack.^74^ All of the above extensions require further investigation and will offer additional insights into the neural basis of migraine.

In summary, we conducted a comprehensive and complex cortical coherence analysis in interictal migraine under different types of stimuli, as well as in resting-state situations. Migraineurs evinced significantly lower cortical coherence of alpha-band neural activities in the frontal clusters during the sensory-evoked recording (auditory and visual stimuli), as well as the resting-state, compared with headache-free controls and multiple factors (such as electrode distance or inter- versus intra-hemispheric signals) appear to modulate the cortical coherence alterations in migraine. The observed abnormalities in desynchronized neural activity across the cortex in this study may be explained by thalamocortical dysrhythmia that has been associated with migraine.^16^^,^^74^ Further studies to identify the underlying mechanisms of cortical coherence and sensory processing abnormalities could lead to improved treatments for migraine patients.

## Supplementary material

Supplementary material is available at *Brain Communications* online.

## Supplementary Material

fcab061_supplementary_data

## Abbreviations

LSD =: least significant difference
PCC =: Pearson’s correlation coefficient
RT =: reaction times
