# Supplementary material for: Abnormalities in cortical pattern of coherence in migraine detected using ultra high-density EEG
Source: Brain Commun. 2021 Apr 2;3(2):fcab061. doi: 10.1093/braincomms/fcab061 (PMC8269966; doi:10.1093/braincomms/fcab061)
Supplement: fcab061_supplementary_data [file fcab061_supplementary_data.pdf]

Supplementary materials for
"Abnormalities in cortical pattern of coherence in migraine detected using ultra high-density EEG"

Short title: Abnormal cortical coherence in migraine

*Alireza Chamanzar[1,2], *Sarah M Haigh[3,4,5], Pulkit Grover[1,2], Marlene Behrmann[2,5]

[1] Department of Electrical and Computer Engineering, Carnegie Mellon University, Pittsburgh, PA, USA.
[2] Neuroscience Institute, Carnegie Mellon University, Pittsburgh, PA, United States.
[3] Department of Psychology, University of Nevada, Reno, NV, United States.
[4] Institute for Neuroscience, University of Nevada, Reno, NV, United States.
[5] Department of Psychology, Carnegie Mellon University, Pittsburgh, PA, United States.

* Co-first authors

Corresponding authors:

Name: Alireza Chamanzar
Address: Hamerschlag Hall B200, 5000 Forbes Ave., Pittsburgh, PA, 15213 United States.
Email: achamanz@andrew.cmu.edu

Name: Sarah M Haigh
Address: 424 Mack Social Sciences, 1664 N. Virginia Street, Reno, NV, 89557 United States.
Email: shaigh@unr.edu

This file includes:

- **Supplementary Note A-D**
- **Supplementary Figure 1-17**
- **Supplementary Table 1**
- **Supplementary References**

Supplementary material:

6Hz stimulation frequency Supplementary Figures:

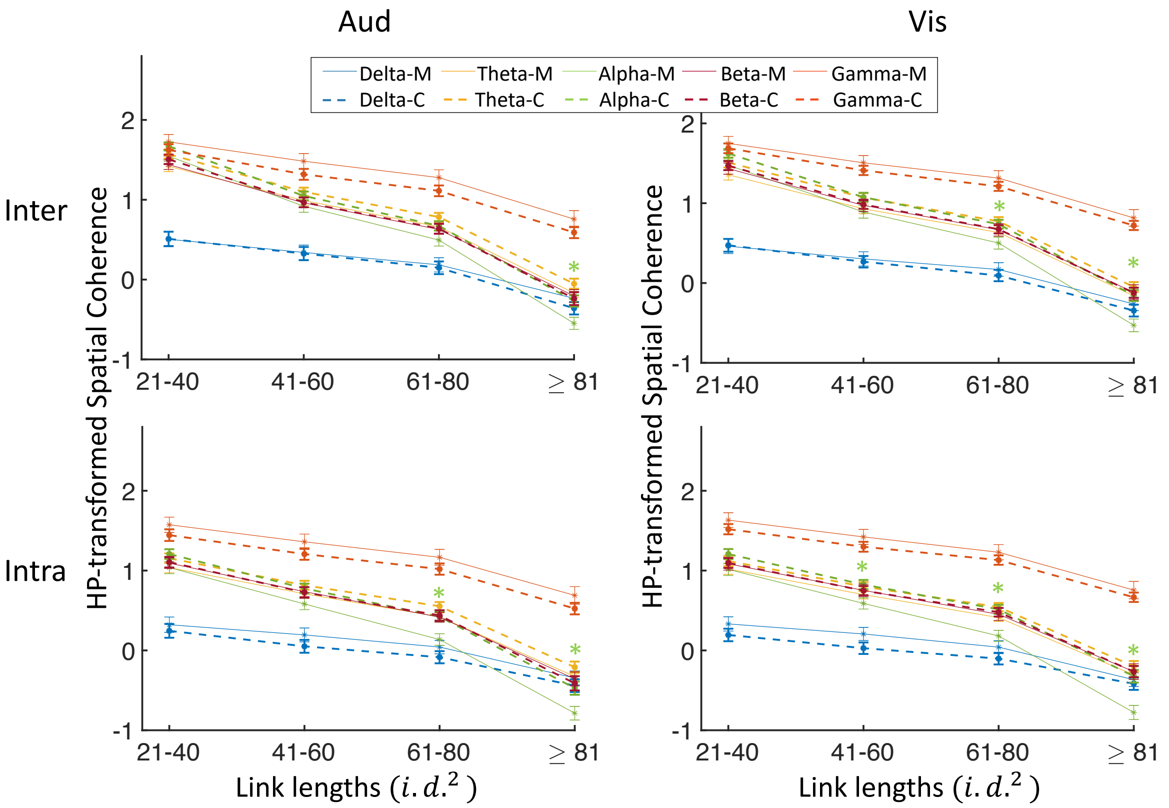

Supplementary Figure 1. Four-way coherence interaction of group x hemisphere x frequency bands x link lengths for 6Hz stimulation frequency. Comparison of spatial coherence between individuals with migraine and controls for the visual and auditory stimulation frequency of 6Hz as a function of link lengths for each of the five frequency bands, each of the hemisphere (inter/intra), and groups. HP-transformed spatial coherence is shown using dashed lines for controls and solid lines for migraineurs. Asterisks show the significant group differences for each link length (on the x-axis) and each frequency band (colors of asterisks are matched with the frequency bands), based on least significant difference (LSD; p<.02) post-hoc test (M= migraineurs, C=controls).

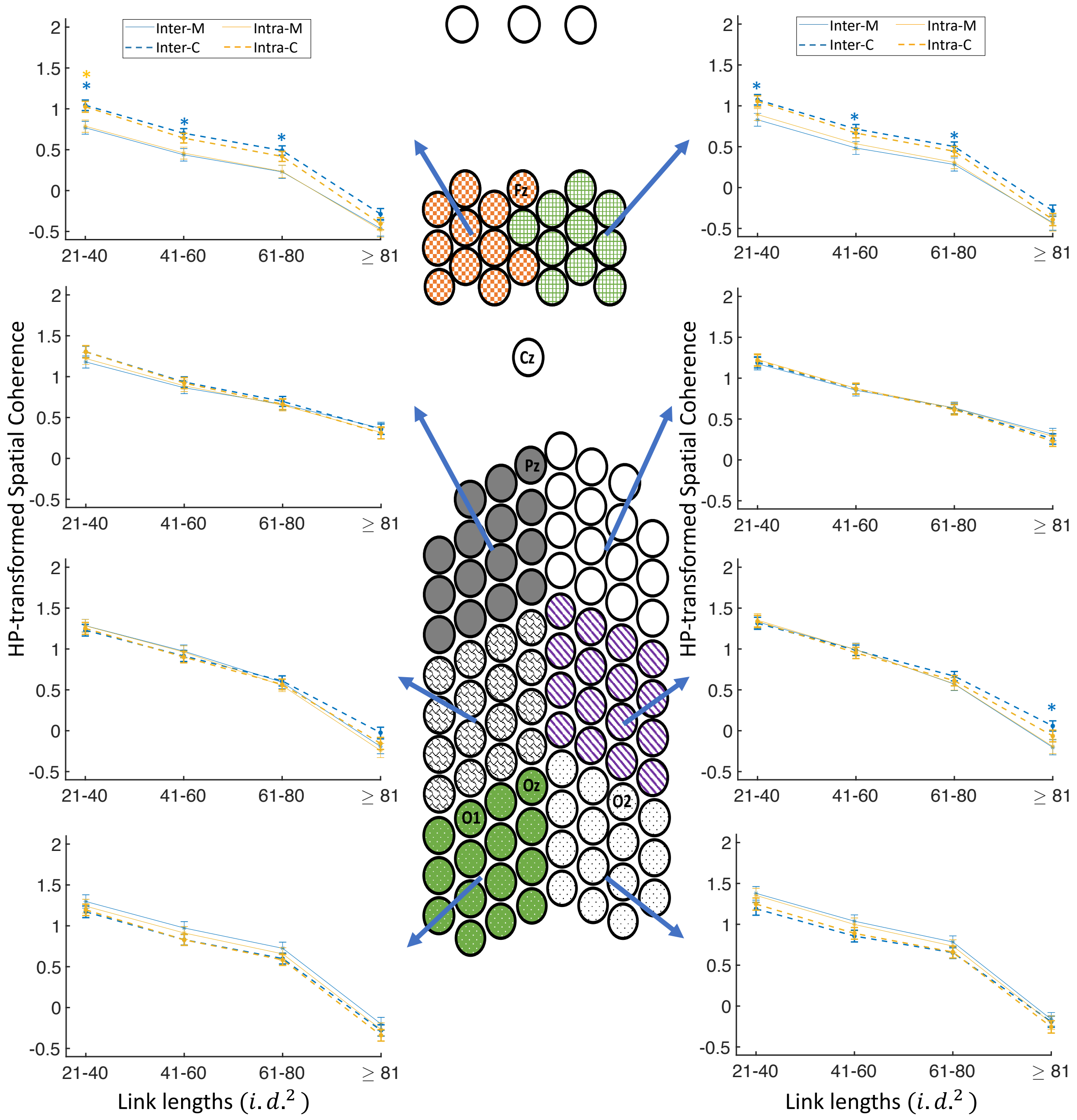

Supplementary Figure 2. Three-way coherence interaction of group x spatial clusters x link lengths for 6Hz stimulation frequency. Comparison of spatial coherence between individuals with migraine and controls for the visual and auditory stimulation frequency of 6Hz as a function of link lengths for each of the eight spatial clusters, and groups. HP-transformed spatial coherence is shown using dashed lines for controls and solid lines for the migraine patients. Asterisks show the significant group differences for each link length (on the x-axis) and each modality (colors of asterisks are matched with the modality), based on least significant difference (LSD; p<.05) post-hoc test (M= migraineurs, C=controls).

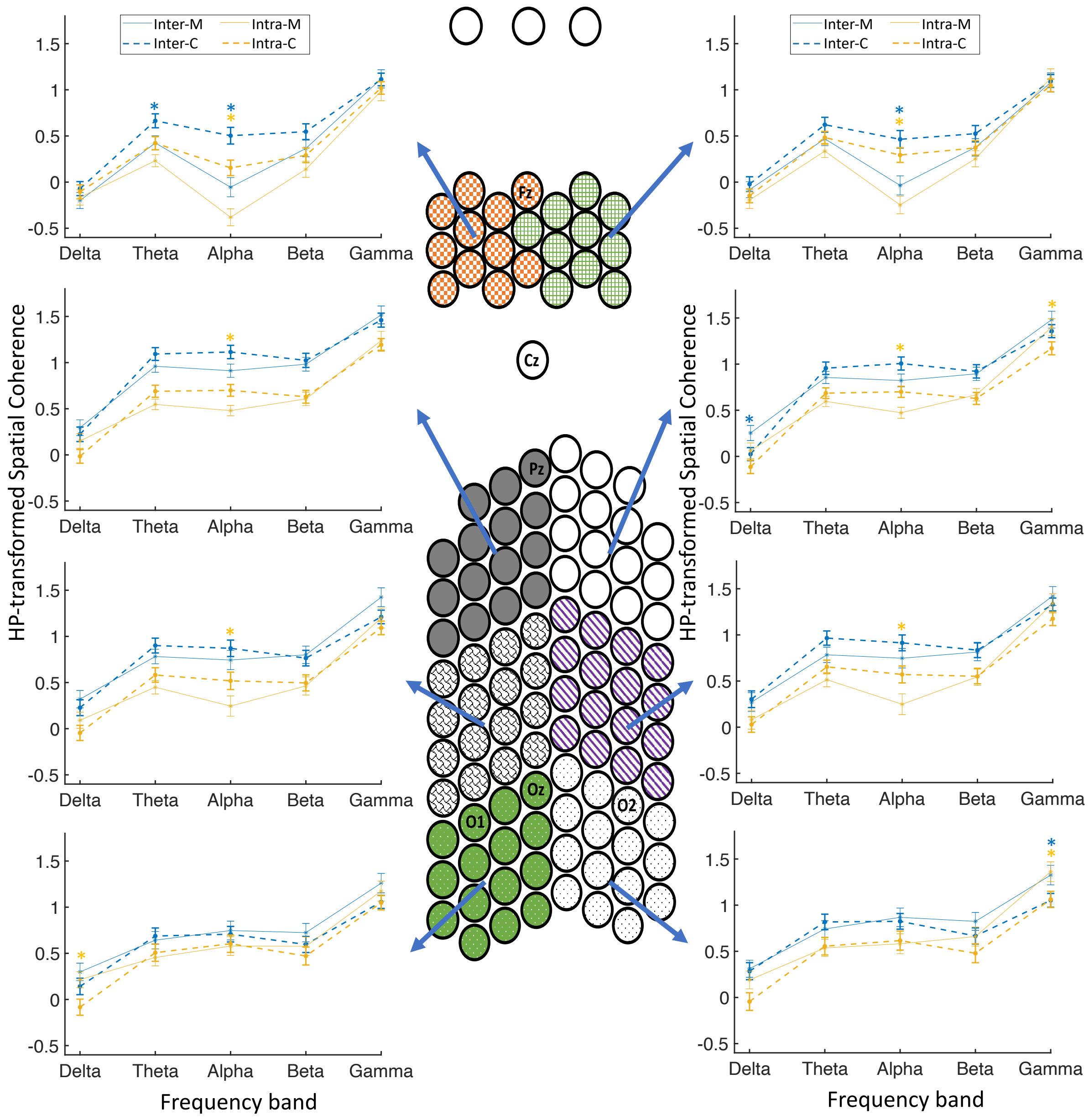

Supplementary Figure 3. Four-way coherence interaction of group x hemisphere x frequency bands x spatial clusters for 6Hz stimulation frequency. Comparison of spatial coherence between individuals with migraine and controls for the stimulation frequency of 6Hz as a function of frequency bands for each of the eight spatial clusters, hemisphere (intra/inter), and groups. HP-transformed spatial coherence is shown using dashed lines for controls and solid lines for migraineurs. Asterisks show the significant group differences for each frequency band (on the x-axis) and each hemisphere (colors of asterisks are matched with the inter- and intra-hemisphere), based on least significant difference (LSD; $p<.05$) post-hoc test (M= migraineurs, C=controls).

Supplementary Note A. Comparison of spatial coherence for migraineurs with aura against their controls:

The spatial coherence analyses in this paper were repeated for the 9 migraine patients with aura in this study, along with their matched control subjects. Note that due to the very small number of migraineurs without aura (only five participants), similar comparison cannot be done for this group. Compared to the results of coherence analysis in Results, where the migraineurs with and without aura were pooled together, the HP-transformed coherence of migraineurs with aura and their controls showed similar significant interactions:

(i) Measures of coherence during visual and auditory stimulation:

The mixed-model ANOVA for 4Hz stimulation frequency revealed a five-way interaction of group x link lengths x frequency bands x hemisphere x spatial clusters ($F(84,1344)=1.29$, $p<.05$), and a three-way interaction of group x frequency bands x modalities ($F(4,64)=2.54$, $p<.05$), along with several lower level interactions which are all subsets of the aforementioned higher-level interactions. The mixed-model ANOVA for 6Hz stimulation frequency revealed a five-way interaction of group x link lengths x frequency bands x hemisphere x spatial clusters ($F(84,1344)=1.38$, $p<.02$), along with several lower level interactions which are all subsets of the aforementioned higher-level interactions.

Post-hoc analysis for the three-way interaction of group x frequency bands x modalities for 4Hz stimulation frequency: Migraineurs with aura showed significantly higher spatial coherence in visual and auditory stimulation frequency of 4Hz in the delta, beta, and gamma frequency bands ($p<.02$; $M=.755\pm.018$, $C=.487\pm.016$), and in visual stimulation frequency of 4Hz in the theta frequency band ($p<.005$; $M=.777\pm.035$, $C=.614\pm.040$). In addition, migraineurs with aura showed significantly lower spatial coherence in auditory stimulation frequency of 4Hz in the alpha frequency band ($p<.05$; $M=.517\pm.044$, $C=.623\pm.036$). Supplementary Fig. 4 shows these group differences.

Post-hoc analysis for the five-way interaction of group x link lengths x frequency bands x hemisphere x spatial clusters for 4Hz stimulation frequency: Migraineurs with aura showed significantly lower spatial coherence in both frontal clusters for stimulation frequency of 4Hz in the alpha frequency band for long intra-hemisphere connections of 61-80 $i.d.^2$($p<.02$; $M=-0.338\pm.147$, $C=.261\pm.111$), in both frontal clusters in the alpha frequency

band for medium-length and long inter-hemisphere connections of $\geq 41\ i.d.^2$(p<.05; M=-0.368$\pm$.090, C=.136$\pm$.083), in left frontal cluster in the theta frequency band for short inter-hemisphere connections of 21-40 $i.d.^2$(p<.03; M=1.117$\pm$.098, C=1.446$\pm$.136), and in both occipito-parietal clusters in the alpha frequency band for long intra-hemisphere connections of $\geq 81\ i.d.^2$(p<.02; M=-1.018$\pm$.144, C=-0.448$\pm$.148). In addition, migraineurs with aura showed significantly higher spatial coherence in right parietal clusters in the delta frequency band for long inter- and intra-hemisphere connections of $\geq 81\ i.d.^2$(p<.05; M=.066$\pm$.152, C=-0.510$\pm$.106), in both occipital cluster in the delta frequency band for intra-hemisphere connections of 41-60 $i.d.^2$(p<.05; M=.557$\pm$.162, C=-0.118$\pm$.140), in both occipital cluster in the beta and alpha frequency band for inter-hemisphere connections of 41-60 $i.d.^2$(p<.05; M=1.362$\pm$.090, C=.865$\pm$.068), and in left occipital cluster in the delta frequency band for long inter-hemisphere connections of $\geq 81\ i.d.^2$(p<.05; M=-0.052$\pm$.229, C=-0.677$\pm$.179), along with few other significant group differences (see Supplementary Fig. 5 and 6 for details).

Post-hoc analysis for the five-way interaction of group x link lengths x frequency bands x hemisphere x spatial clusters for 6Hz stimulation frequency: Migraineurs with aura showed significantly lower spatial coherence in both frontal and both occipito-parietal clusters for stimulation frequency of 6Hz in the alpha frequency band for long inter- and intra-hemisphere connections of $\geq 81\ i.d.^2$(p<.02; M=-1.076$\pm$.056, C=-0.531$\pm$.054), and in left frontal cluster in the theta frequency band for short and long inter-hemisphere connections of 21-40 and 61-80 $i.d.^2$(p<.05; M=.783$\pm$.088, C=1.050$\pm$.083), along with few other significant group differences (see Supplementary Fig. 7 and 8 for details).

(ii) Measures of coherence during resting-state recordings:

The mixed-model ANOVA for resting-state recording revealed a three-way interaction of group x link lengths x spatial clusters (F(21,336)=2.10, p<.01), along with several lower level interactions which are all subsets of the aforementioned higher-level interactions. In comparison with controls, migraineurs showed significantly higher spatial coherence in both occipital clusters for link lengths of $\geq 21\ i.d.^2$(p<.04; M=.900$\pm$.041, C=.563$\pm$.031), and in right parietal clusters for link lengths of $\geq 41\ i.d.^2$(p<.05; M=.800$\pm$.056, C=.520$\pm$.041), and in right occipito-parietal cluster for link length of 41-60 $i.d.^2$(p<.03; M=1.151$\pm$.099, C=.867$\pm$.069). Unlike the resting-state results of coherence analysis in Results (migraineurs with and without aura were pooled together), there was no

frequency band factor in this three-way interaction. However, looking at each frequency band individually, migraineurs with aura and their controls showed similar patterns of group difference in the right frontal clusters for alpha frequency band (lower coherence in migraine), and parietal and occipital clusters for beta and gamma frequency bands (higher coherence in migraine). For the results of resting-state coherence analysis, see Supplementary Fig. 9 for migraineurs with aura, and Fig. 7 for migraineurs with and without aura pooled together.

In summary, compared to the sensory stimulation and resting-state results of coherence analysis in Results (migraineurs with and without aura were pooled together), migraineurs with aura showed mostly consistent patterns of group differences, i.e., the frontal clusters in migraineurs with aura showed significant lower spatial coherence in the alpha frequency band.

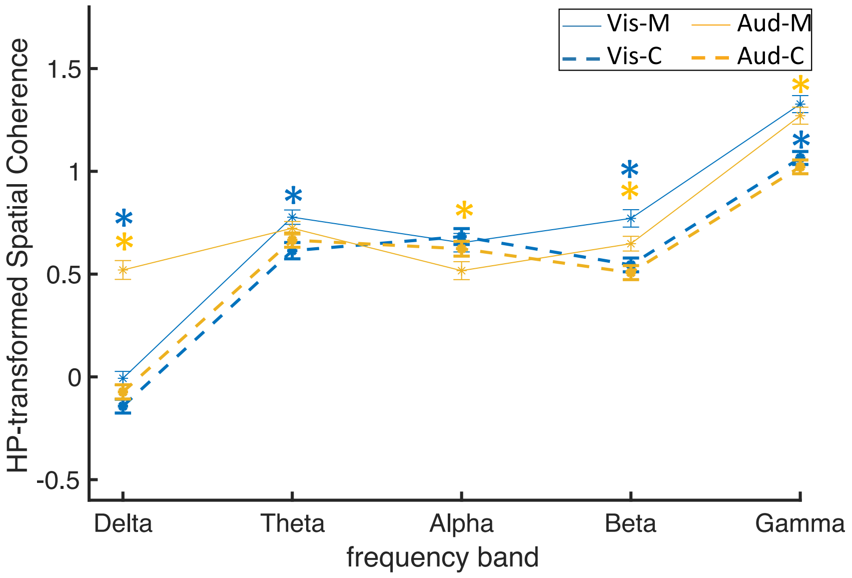

Supplementary Figure 4. Three-way coherence interaction of group x frequency bands x modalities for 4Hz stimulation frequency in migraineurs with aura and controls. Comparison of spatial coherence between migraineurs with aura and controls for the stimulation frequency of 4Hz as a function of frequency bands for each of the modalities, and groups. HP-transformed spatial coherence is shown using dashed lines for controls and solid lines for migraineurs. Asterisks show the significant group differences for each frequency band (on the x-axis) and each modality (colors of asterisks are matched with the visual and auditory modalities), based on least significant difference (LSD; $p<.05$) post-hoc test (M= migraineurs, C=controls).

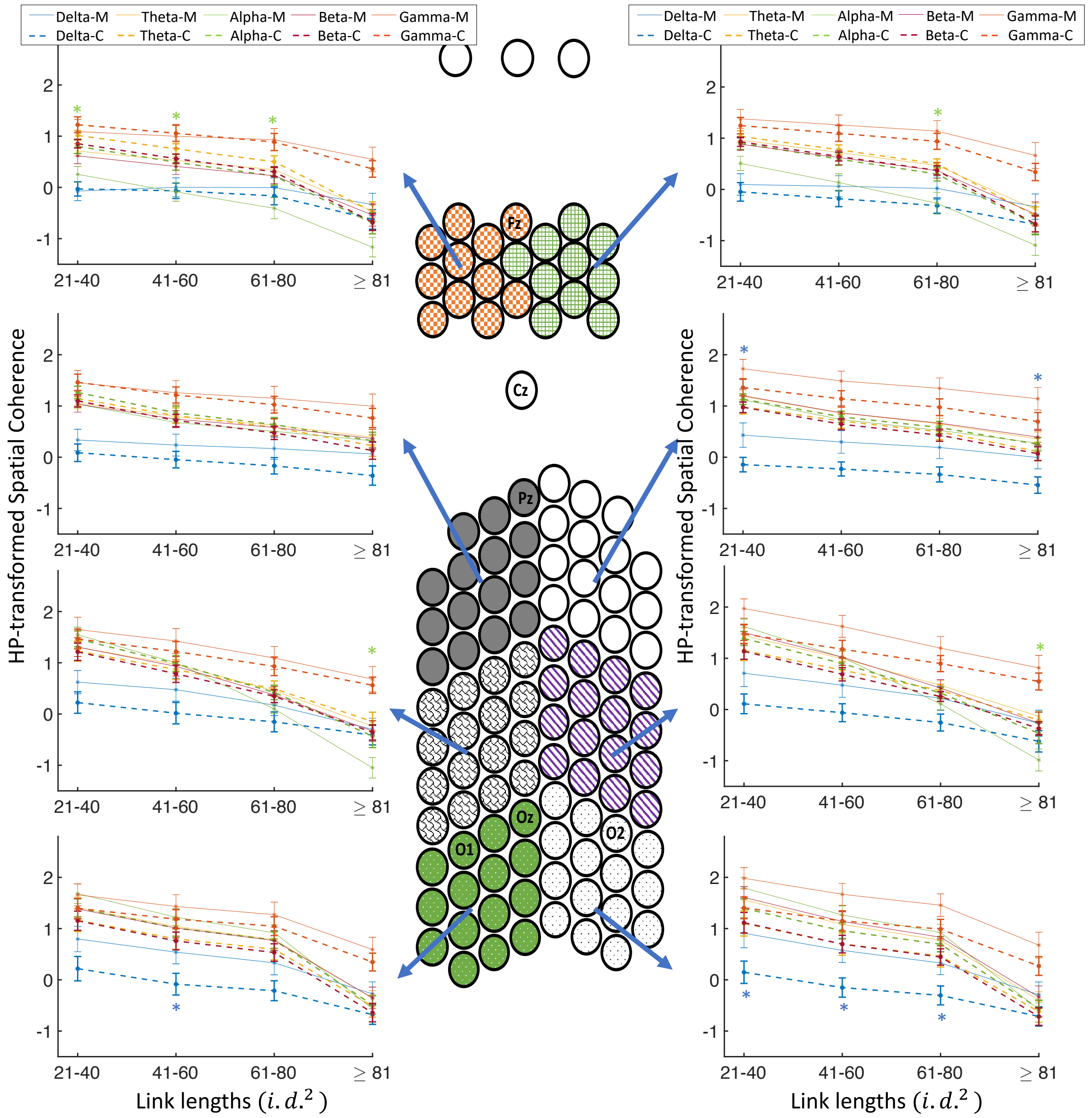

Supplementary Figure 5. Five-way interaction of group x link lengths x frequency bands x hemisphere x spatial clusters for 4Hz stimulation frequency in migraineurs with aura and controls. Comparison of spatial coherence between migraineurs with aura and controls for the stimulation frequency of 4Hz for intra-hemisphere connections, as a function of link lengths for each of the five frequency bands, each of the eight spatial clusters, and groups (Supplementary Fig. 6 shows the results for inter-hemisphere connections). HP-transformed spatial coherence is shown using dashed lines for controls and solid lines for migraineurs. Asterisks show the significant group differences for each link length (on the x-axis) and each frequency band (colors of asterisks are matched with the frequency bands), based on least significant difference (LSD; p<.05) post-hoc test (M= migraineurs, C=controls).

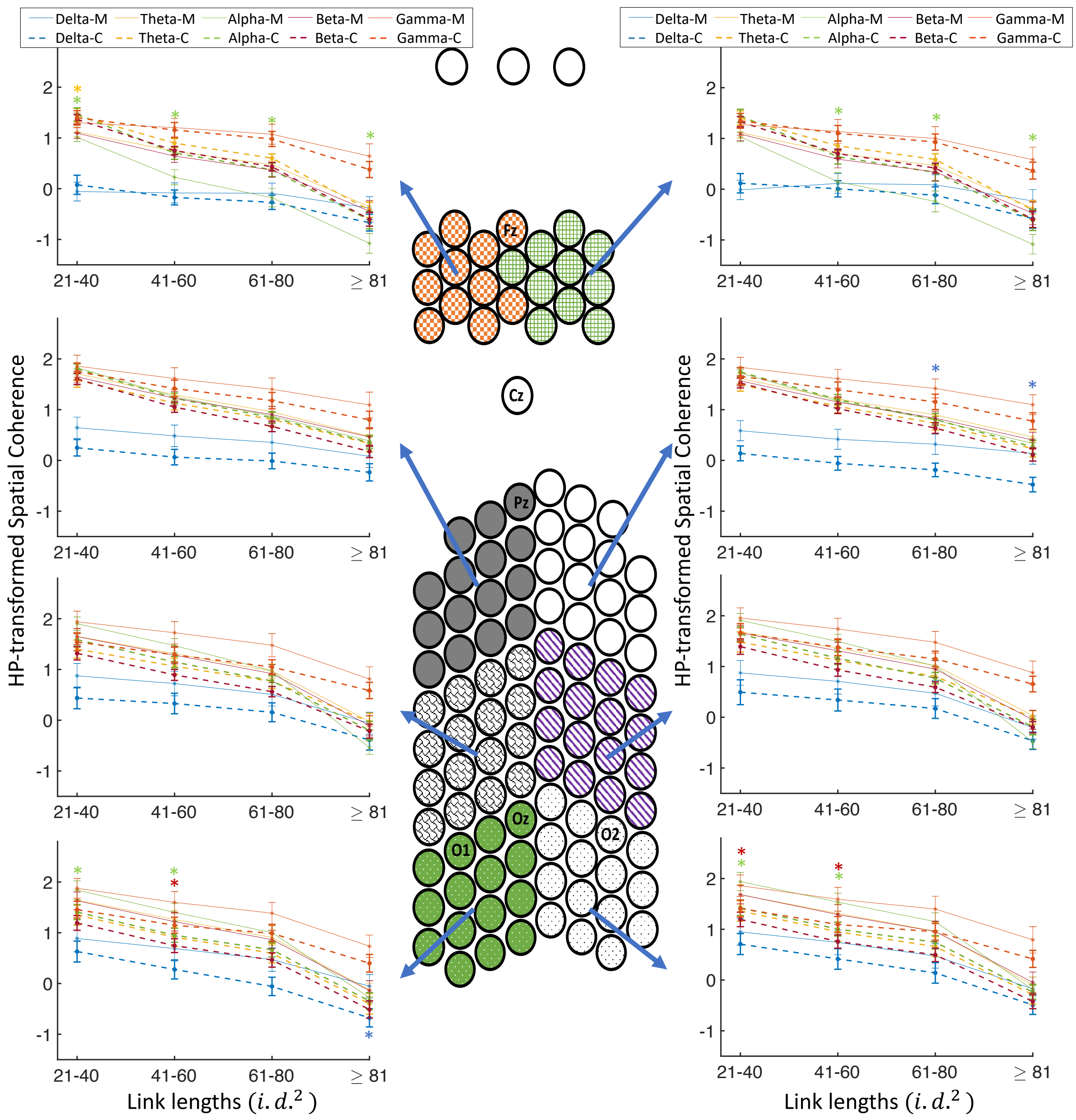

Supplementary Figure 6. Five-way interaction of group x link lengths x frequency bands x hemisphere x spatial clusters for 4Hz stimulation frequency in migraineurs with aura and controls. Comparison of spatial coherence between migraineurs with aura and controls for the stimulation frequency of 4Hz for inter-hemisphere connections, as a function of link lengths for each of the five frequency bands, each of the eight spatial clusters, and groups (Supplementary Fig. 5 shows the results for intra-hemisphere connections). HP-transformed spatial coherence is shown using dashed lines for controls and solid lines for migraineurs. Asterisks show the significant group differences for each link length (on the x-axis) and each frequency band (colors of asterisks are matched with the frequency bands), based on least significant difference (LSD; p<.05) post-hoc test (M= migraineurs, C=controls).

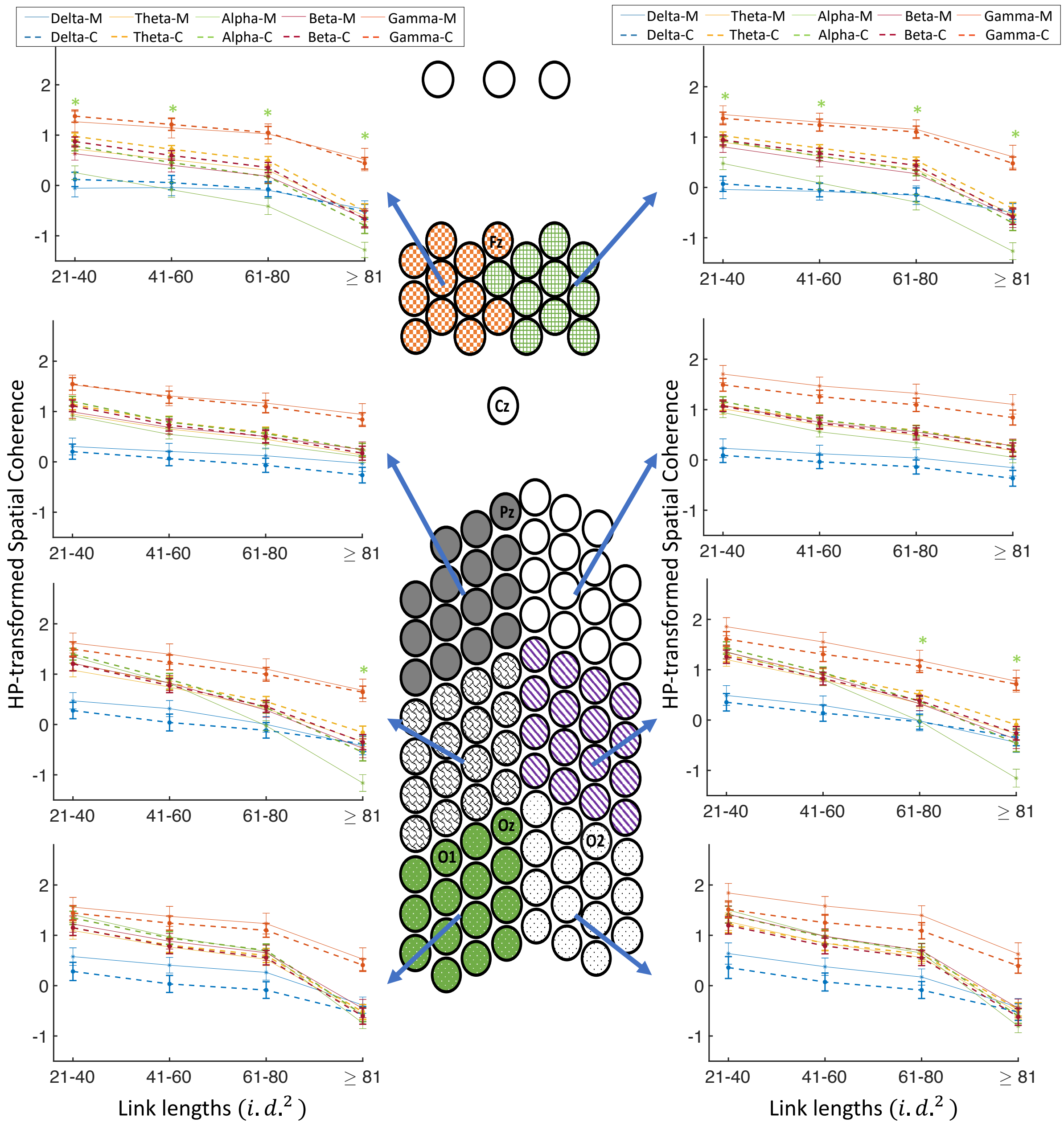

Supplementary Figure 7. Five-way interaction of group x link lengths x frequency bands x hemisphere x spatial clusters for 6Hz stimulation frequency in migraineurs with aura and controls. Comparison of spatial coherence between migraineurs with aura and controls for the stimulation frequency of 6Hz for intra-hemisphere connections, as a function of link lengths for each of the five frequency bands, each of the eight spatial clusters, and groups (Supplementary Fig. 8 shows the results for inter-hemisphere connections). HP-transformed spatial coherence is shown using dashed lines for controls and solid lines for migraineurs. Asterisks show the significant group differences for each link length (on the x-axis) and each frequency band (colors of asterisks are matched with the frequency bands), based on least significant difference (LSD; $p<.05$) post-hoc test (M= migraineurs, C=controls).

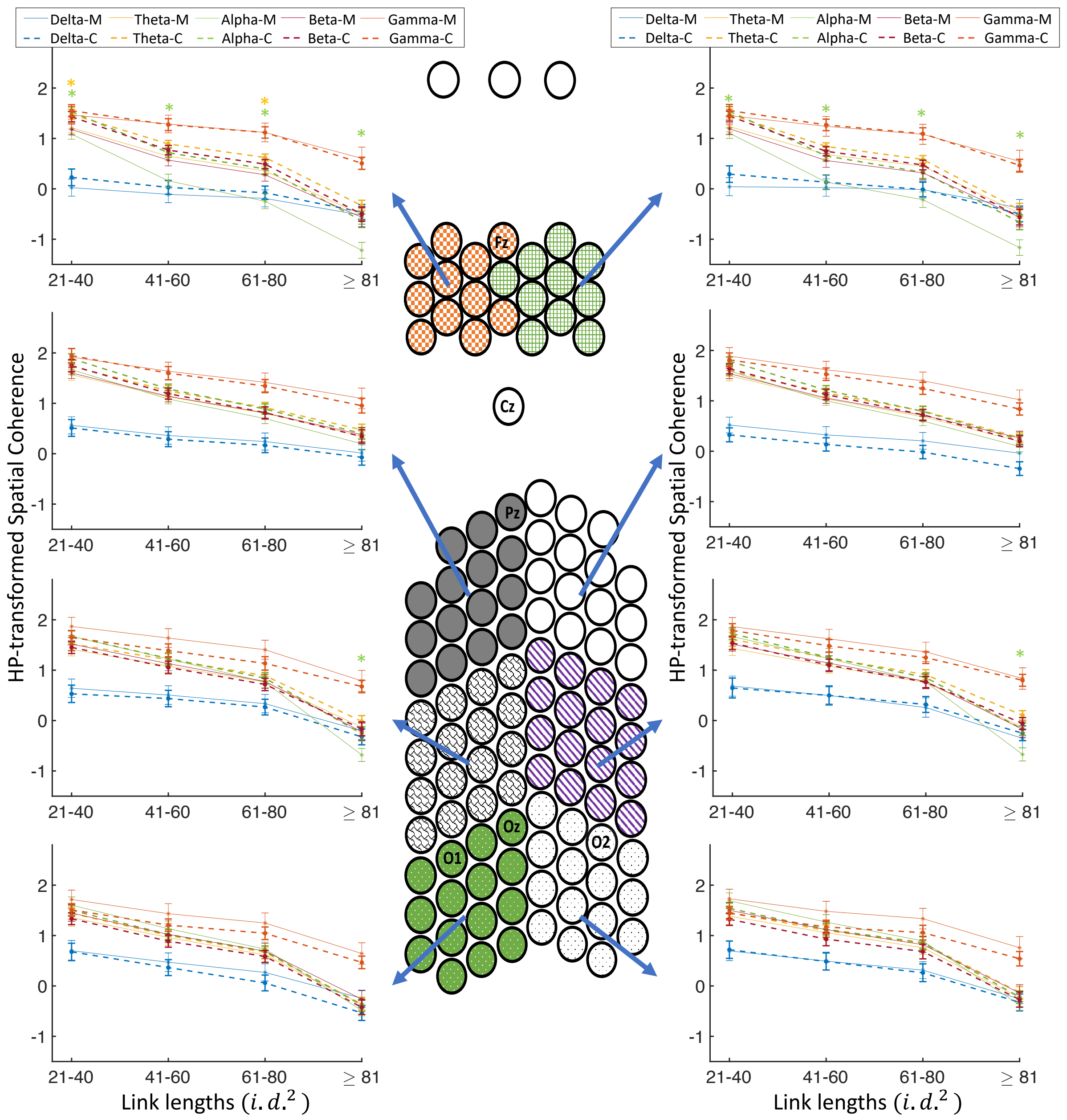

Supplementary Figure 8. Five-way interaction of group x link lengths x frequency bands x hemisphere x spatial clusters for 6Hz stimulation frequency in migraineurs with aura and controls. Comparison of spatial coherence between migraineurs with aura and controls for the stimulation frequency of 6Hz for inter-hemisphere connections, as a function of link lengths for each of the five frequency bands, each of the eight spatial clusters, and groups (Supplementary Fig. 7 shows the results for intra-hemisphere connections). HP-transformed spatial coherence is shown using dashed lines for controls and solid lines for migraineurs. Asterisks show the significant group differences for each link length (on the x-axis) and each frequency band (colors of asterisks are matched with the frequency bands), based on least significant difference (LSD; $p<.05$) post-hoc test (M= migraineurs, C=controls).

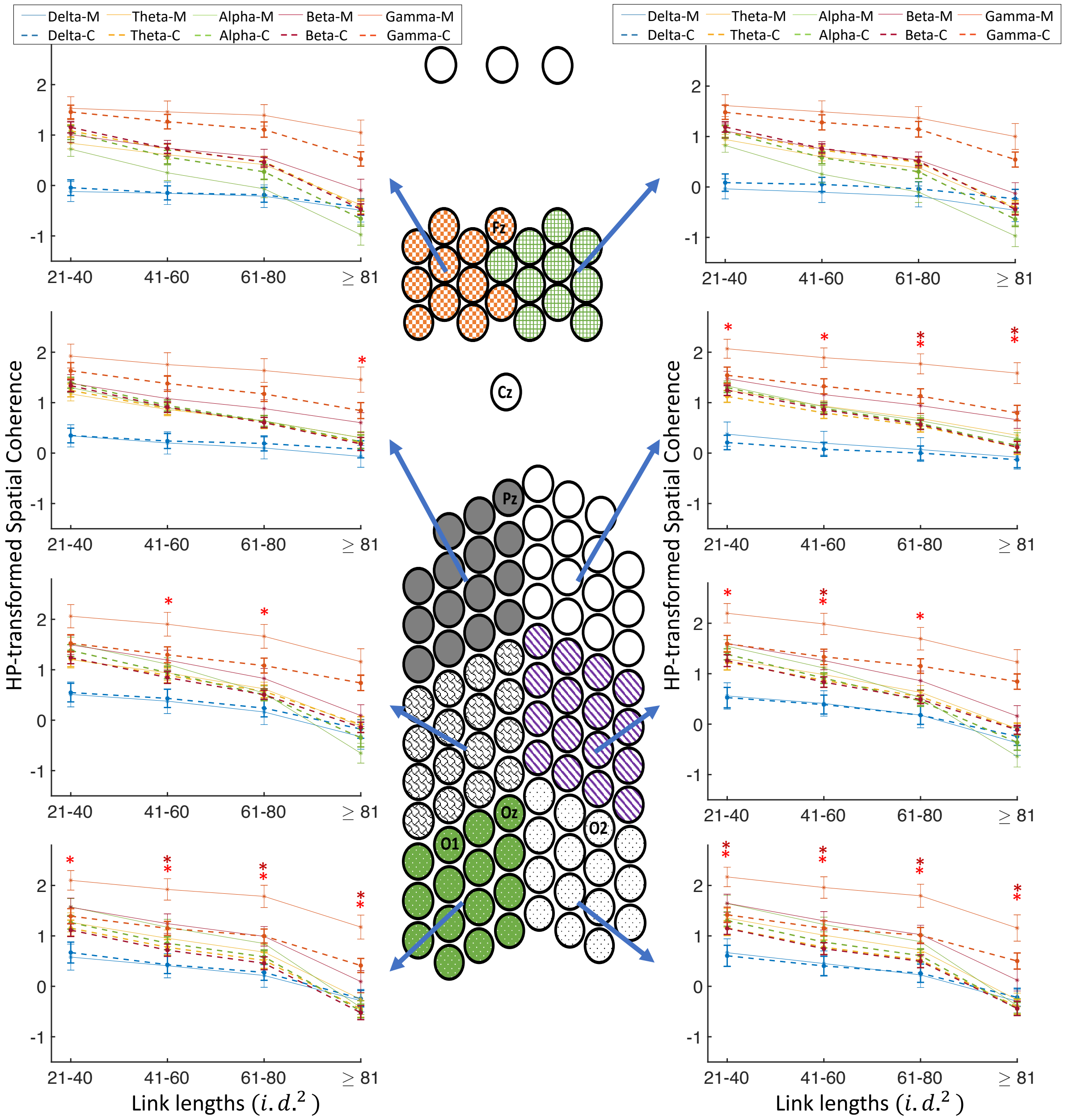

Supplementary Figure 9. Three-way resting-state coherence interaction of group x spatial clusters x link lengths for different frequency bands in migraineurs with aura and controls.. Comparison of spatial coherence between migraineurs with aura and controls during the resting-state recording as a function of link lengths for each of the five frequency bands, each of the eight spatial clusters, and groups. HP-transformed spatial coherence is shown using dashed lines for controls and solid lines for the migraine patients. Asterisks show the significant group differences for each link length (on the x-axis) and each frequency band (colors of asterisks are matched with the frequency bands), based on least significant difference (LSD; p<.05) post-hoc test (M= migraineurs, C=controls).

Supplementary Note B. Coherence normalization:

Because the absolute value of spatial coherence has different ranges of values for different inter-electrode distances (small values for long links and vice versa), we repeated the spatial coherence analysis using the normalized coherence values to confirm that the reported results are not affected by this potential confound. The exponential function ($ae^{bx} + c$) which was fitted to the averaged absolute value of PCCs was found to be $a = 0.63, b = -8.7 \times 10^{-3}, c = 0.23$, with 95% confidence interval of $a \in [0.62,0.64]$, $b \in [-9.0, -8.3] \times 10^{-3}$, and $c \in [0.220,0.234]$. Supplementary Fig. 10 shows the averaged absolute value of PCCs and the fitted curve as functions of inter-electrode distances (squared Euclidean distance in $i.d.^2$, where $i.d.$ is the distance unit in 2D map of Fig. 2).

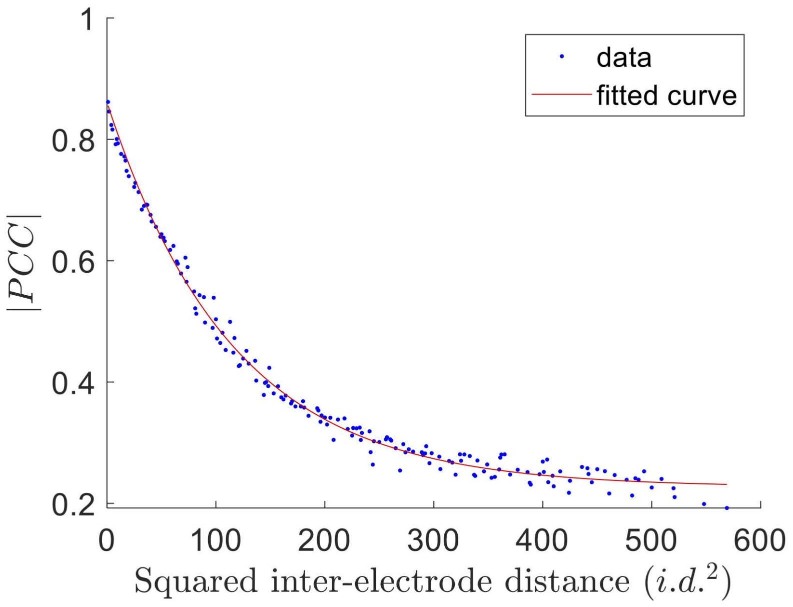

Supplementary Figure 10. Coherence normalization. An exponential function was used (red curve) to fit to the averaged absolute value of PCCs (blue dots) as functions of inter-electrode distances (squared Euclidean distance in $i.d.^2$, where $i.d.$ is the distance unit in 2D map of Fig. 2).

All absolute values of PCCs are divided by this function at each inter-electrode distance. Then the normalized PCCs are HP-transformed (see the Materials and methods, Statistical analysis subsection, for more details on using HP transform to correct for the violation of normality and homoscedasticity assumptions). The spatial coherence analyses in this paper were repeated using the normalized and HP-transformed PCCs. Compared to the

results in Results, Coherence subsection, the normalized coherence showed similar significant interactions:

The mixed-model ANOVA for 4Hz stimulation frequency revealed a five-way interaction of group x link lengths x frequency bands x hemisphere x modalities ($F(12,312)=2.45$, $p<.01$), a four-way interaction of group x hemisphere x frequency bands x spatial clusters ($F(28,728)=1.55$, $p<.04$), and a four-way interaction of group x link lengths x modalities x spatial clusters ($F(21,546)=1.62$, $p<.04$), along with several lower level interactions which are all subsets of the aforementioned higher-level interactions.

The mixed-model ANOVA for 6Hz stimulation frequency revealed similar interactions: a five-way interaction of group x link lengths x frequency bands x hemisphere x modalities ($F(12,312)=1.86$, $p<.04$), a four-way interaction of group x hemisphere x frequency bands x spatial clusters ($F(28,728)=1.71$, $p<.02$), and a three-way interaction of group x link lengths x spatial clusters ($F(21,546)=1.67$, $p<.03$), along with several lower level interactions which are all subsets of the aforementioned higher-level interactions. However, unlike unnormalized coherence, the hemisphere (inter/intra) factor did not show up in the four-way interaction of group x link lengths x modalities x spatial clusters for 4Hz stimulation frequency, and there was an additional factor of modalities in the five-way interaction of group x link lengths x frequency bands x hemisphere x modalities for both stimulation frequencies (4Hz and 6Hz).

Supplementary Fig. 11 shows the normalized and HP-transformed spatial coherence values for stimulation frequency of 4Hz (visual and auditory separately) as a function of link lengths for each of the five frequency bands, hemisphere (intra/inter), and groups (the five-way interaction of group x link lengths x frequency bands x hemisphere x modalities). Based on the results, there were no group differences between migraineurs and headache-free controls (although the trend is in the same direction in comparison with the results of the unnormalized coherence analysis).

Supplementary Fig. 12 shows the spatial coherence values for stimulation frequency of 6Hz (visual and auditory separately) as a function of link lengths for each of the five frequency bands, hemisphere (intra/inter), and groups (the five-way interaction of group x link lengths x frequency bands x hemisphere x modalities). In comparison with controls, migraineurs showed significantly lower spatial coherence for visual stimulation frequency of 6Hz in the alpha frequency band for long inter- and intra-hemisphere connections of ≥ 81 $i.d.^2$($p<.03$;

M=.810$\pm$.084, C=1.117$\pm$.077), significantly higher spatial coherence for visual stimulation frequency of 6Hz in the delta frequency band for intra-hemisphere connections of 61-80 $i.d.^2$(p<.05; M=.522$\pm$.104, C=.224$\pm$.105), and higher spatial coherence for auditory stimulation frequency of 6Hz in the beta frequency band for long intra-hemisphere connections of ≥81 $i.d.^2$(p<.05; M=.890$\pm$.125, C=.637$\pm$.130).

To summarize, similar to the unnormalized coherence, migraineurs showed significantly lower spatial coherence in the alpha frequency band for long inter-electrode distances during the visual stimuli for 6Hz stimulation frequency (but not for 4Hz stimulation frequency, although the trend is in the same direction).

Supplementary Fig. 13 shows the spatial coherence values for stimulation frequency of 4Hz as a function of frequency bands for each of the eight spatial clusters, hemisphere (intra/inter), and groups (the four-way interaction of group x hemisphere x frequency bands x spatial clusters). Based on the LSD results for 4Hz stimulation frequency, compared with controls, migraineurs showed significantly lower spatial coherence in the frontal clusters for the alpha frequency band for both inter- and intra-hemisphere connections ($p<10^{-6}$; M=.058$\pm$.047, C=.725$\pm$.040), and in the left frontal cluster for the theta frequency band for inter-hemisphere connections (p<.04; M=.823$\pm$.057, C=1.068$\pm$.060). In addition, migraineurs showed significantly higher spatial coherence in the right parietal and occipital clusters for the gamma frequency band for intra-hemisphere connections (p<.05; M=1.808$\pm$.072, C=1.514$\pm$.063), in the right occipital cluster for the delta and beta frequency band for intra-hemisphere connections (p<.03; M=.858$\pm$.083, C=.488$\pm$.090), and in the right parietal cluster for the delta frequency band for inter-hemisphere connections (p<.01; M=.727$\pm$.094, C=.463$\pm$.082).

Supplementary Fig. 14 shows the spatial coherence values for stimulation frequency of 6Hz as a function of frequency bands for each of the eight spatial clusters, hemisphere (intra/inter), and groups (the four-way interaction of group x hemisphere x frequency bands x spatial clusters). Based on the LSD results for 6Hz stimulation frequency, similar trends of group differences were observed in comparison with the results for 4Hz stimulation frequency: compared with controls, migraineurs showed significantly lower spatial coherence in the frontal clusters for the alpha frequency band for both inter- and intra-hemisphere connections (p<0.0001; M=.063$\pm$.045, C=.671$\pm$.041), and in the left frontal cluster for the theta frequency

band for inter-hemisphere connections ($p<.03$; M=.812$\pm$.061, C=1.064$\pm$.058). In addition, migraineurs showed significantly higher spatial coherence in the right parietal and occipital clusters for the gamma frequency band for intra-hemisphere connections ($p<.05$; M=1.834$\pm$.073, C=1.556$\pm$.063), in the right occipital cluster for the delta and beta frequency band for intra-hemisphere connections ($p<.03$; M=.863$\pm$.082, C=.437$\pm$.090), in the right parietal cluster for the delta frequency band for inter- and intra-hemisphere connections ($p<.03$; M=.572$\pm$.065, C=.295$\pm$.059), in the left parietal cluster for the delta frequency band for intra-hemisphere connections ($p<.01$; M=.759$\pm$.119, C=.263$\pm$.129), and in the left occipital cluster for the delta frequency band for inter- and intra-hemisphere connections ($p<.03$; M=.698$\pm$.064, C=.400$\pm$.063).

In other words, migraineurs showed significant lower inter- and intra-hemisphere coherence in the frontal clusters and in the alpha frequency band, regardless of the modality and stimulation frequency. This result is consistent with the result of unnormalized coherence analysis.

Supplementary Fig. 15 shows the spatial coherence values for visual and auditory stimulation frequency of 4Hz as a function of link lengths for each of the eight spatial clusters, and groups (the four-way interaction of group x link lengths x spatial clusters x modalities). In comparison with controls, migraineurs showed significantly lower spatial coherence for visual stimulation frequency of 4Hz in both frontal clusters for link lengths of ≥21 $i.d.^2$($p<.04$; M=.629$\pm$.034, C=.943$\pm$.028).

Supplementary Fig. 16 shows the spatial coherence values for stimulation frequency of 6Hz as a function of link lengths for each of the visual and auditory modalities, for each of the eight spatial clusters, and groups (the three-way interaction of group x link lengths x spatial clusters). Similar to the results for 4Hz stimulation frequency, using the less conservative LSD test, in comparison with controls, migraineurs showed significantly lower spatial coherence for visual stimulation frequency of 6Hz in both frontal clusters for medium-length and long connections of 41-80 $i.d.^2$($p<.02$; M=.629$\pm$.041, C=.948$\pm$.032), in the right frontal cluster for short links of 21-40 $i.d.^2$($p<.03$; M=.428$\pm$.083, C=.721$\pm$.061), and in the left frontal cluster for long connections of ≥81 $i.d.^2$($p<.02$; M=.747$\pm$.140, C=1.035$\pm$.121). To summarize, similar to the results for unnormalized coherence, migraineurs showed significantly lower

spatial coherence in frontal clusters for medium and long inter-electrode distances during the visual stimuli (but not auditory stimuli, although the trend is in the same direction).

We also conducted the same analysis on the resting-state EEG data. Compared to the unnormalized coherence, there was the same four-way interaction of group x frequency bands x spatial clusters x link length (F(84,2184)=1.49, p<.005) along with several lower level interactions which are all subsets of the aforementioned higher level interaction. Based on the results (see Supplementary Fig. 17), using a less conservative LSD test, in comparison with controls, migraineurs showed significantly lower spatial coherence in both frontal clusters for the alpha frequency band for all link lengths of ≥41 $i.d.^2$(p<.03; M=.070$\pm$.077, C=.645$\pm$.068), in the right frontal cluster for the alpha frequency band for the short links of 21-40 $i.d.^2$(p<.04; M=.185$\pm$.174, C=.700$\pm$.106), in the right frontal cluster for the delta frequency band for the longest connections (p<.03; M=.526$\pm$.260, C=1.118$\pm$.229), and in both frontal clusters for the theta frequency band for the longest connections (p<.02; M=.425$\pm$.124, C=.838$\pm$.130). In addition, in both occipital clusters, migraineurs showed significant higher coherence in the beta frequency band for the longest connections (p<.04; M=1.472$\pm$.168, C=.963$\pm$.155), and in the right parietal cluster for the beta and gamma frequency bands for the longest connections (p<.03; M=1.897$\pm$.158, C=1.434$\pm$.132). These resting-state results are largely consistent with unnormalized coherence analyses (see Supplementary Fig. 17 and Fig. 7).

Taken together, the results of normalized coherence are remarkably similar to those of the unnormalized results with the same preponderance of differences in frontal clusters, and in the alpha band frequency, and largely independent of modality and independent of stimulation frequency.

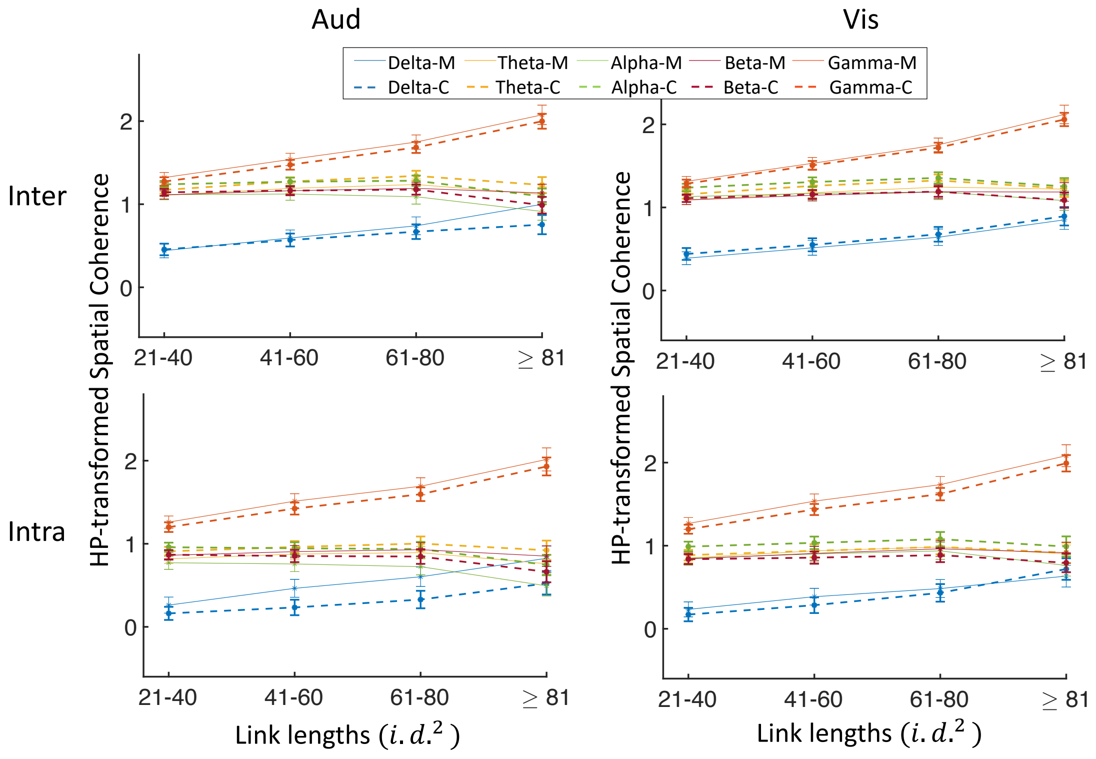

Supplementary Figure 11. Five-way normalized coherence interaction of group x hemisphere x frequency bands x link lengths x modalities for 4Hz stimulation frequency. Comparison of spatial coherence between individuals with migraine and controls for the visual and auditory stimulation frequency of 4Hz as a function of link lengths for each of the five frequency bands, each of the hemisphere (inter/intra), and groups. HP-transformed normalized spatial coherence is shown using dashed lines for controls and solid lines for migraineurs. Asterisks show the significant group differences for each link length (on the x-axis) and each frequency band (colors of asterisks are matched with the frequency bands), based on least significant difference (LSD) post-hoc test (M= migraineurs, C=controls).

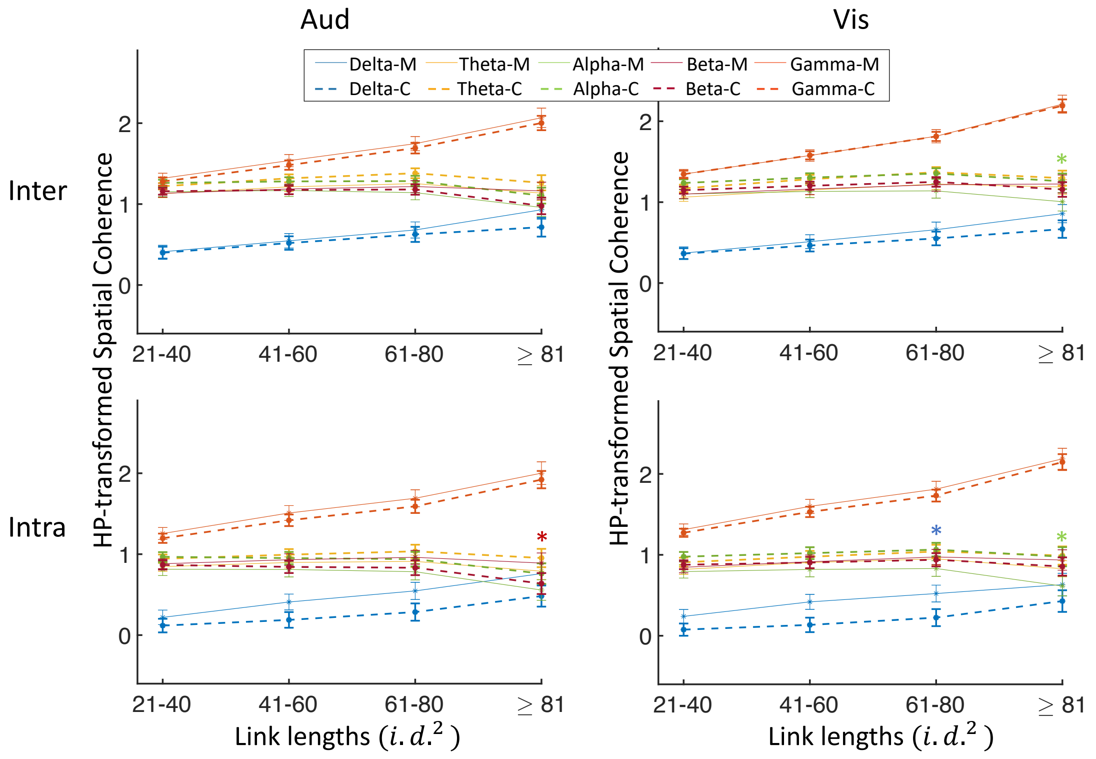

Supplementary Figure 12. Five-way normalized coherence interaction of group x hemisphere x frequency bands x link lengths x modalities for 6Hz stimulation frequency. Comparison of spatial coherence between individuals with migraine and controls for the visual and auditory stimulation frequency of 6Hz as a function of link lengths for each of the five frequency bands, each of the hemisphere (inter/intra), and groups. HP-transformed normalized spatial coherence is shown using dashed lines for controls and solid lines for migraineurs. Asterisks show the significant group differences for each link length (on the x-axis) and each frequency band (colors of asterisks are matched with the frequency bands), based on least significant difference (LSD; $p<.05$) post-hoc test (M= migraineurs, C=controls).

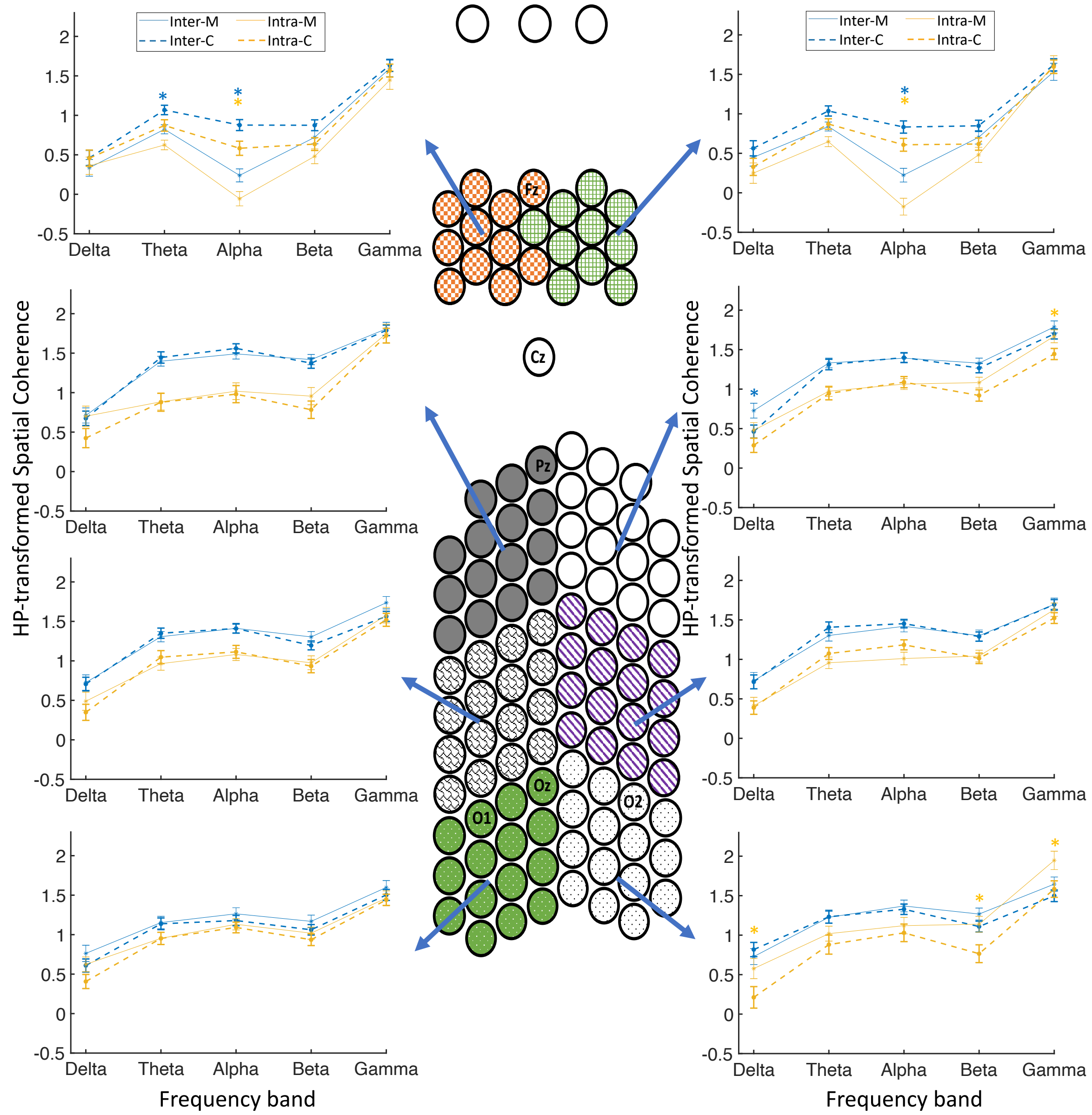

Supplementary Figure 13. Four-way normalized coherence interaction of group x hemisphere x frequency bands x spatial clusters for 4Hz stimulation frequency. Comparison of spatial coherence between individuals with migraine and controls for the stimulation frequency of 4Hz as a function of frequency bands for each of the eight spatial clusters, hemisphere (intra/inter), and groups. HP-transformed normalized spatial coherence is shown using dashed lines for controls and solid lines for migraineurs. Asterisks show the significant group differences for each frequency band (on the x-axis) and each hemisphere (colors of asterisks are matched with the inter- and intra-hemisphere), based on least significant difference (LSD; p<.05) post-hoc test (M= migraineurs, C=controls).

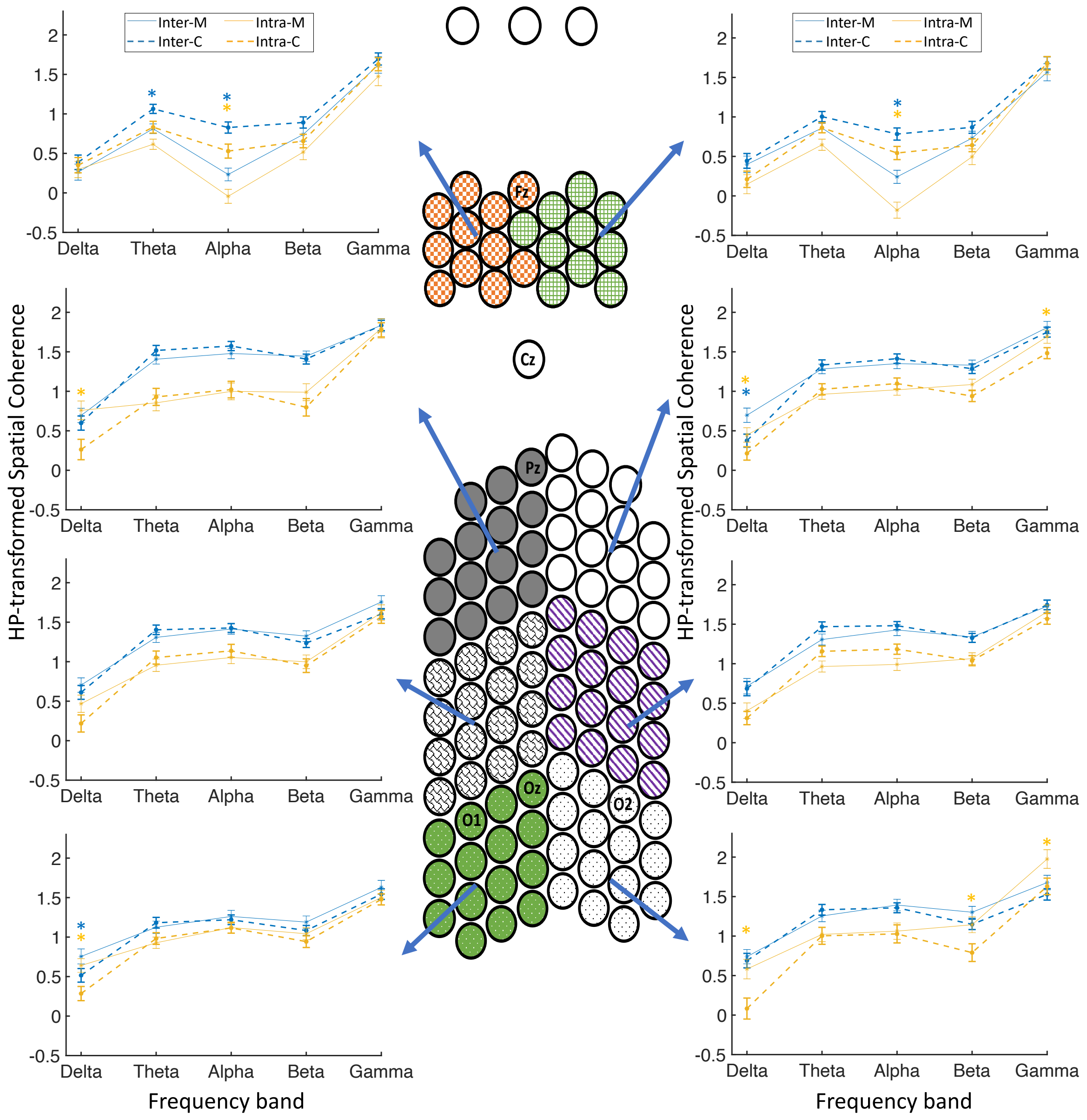

Supplementary Figure 14. Four-way normalized coherence interaction of group x hemisphere x frequency bands x spatial clusters for 6Hz stimulation frequency. Comparison of spatial coherence between individuals with migraine and controls for the stimulation frequency of 6Hz as a function of frequency bands for each of the eight spatial clusters, hemisphere (intra/inter), and groups. HP-transformed normalized spatial coherence is shown using dashed lines for controls and solid lines for migraineurs. Asterisks show the significant group differences for each frequency band (on the x-axis) and each hemisphere (colors of asterisks are matched with the inter- and intra-hemisphere), based on least significant difference (LSD; p<.05) post-hoc test (M= migraineurs, C=controls).

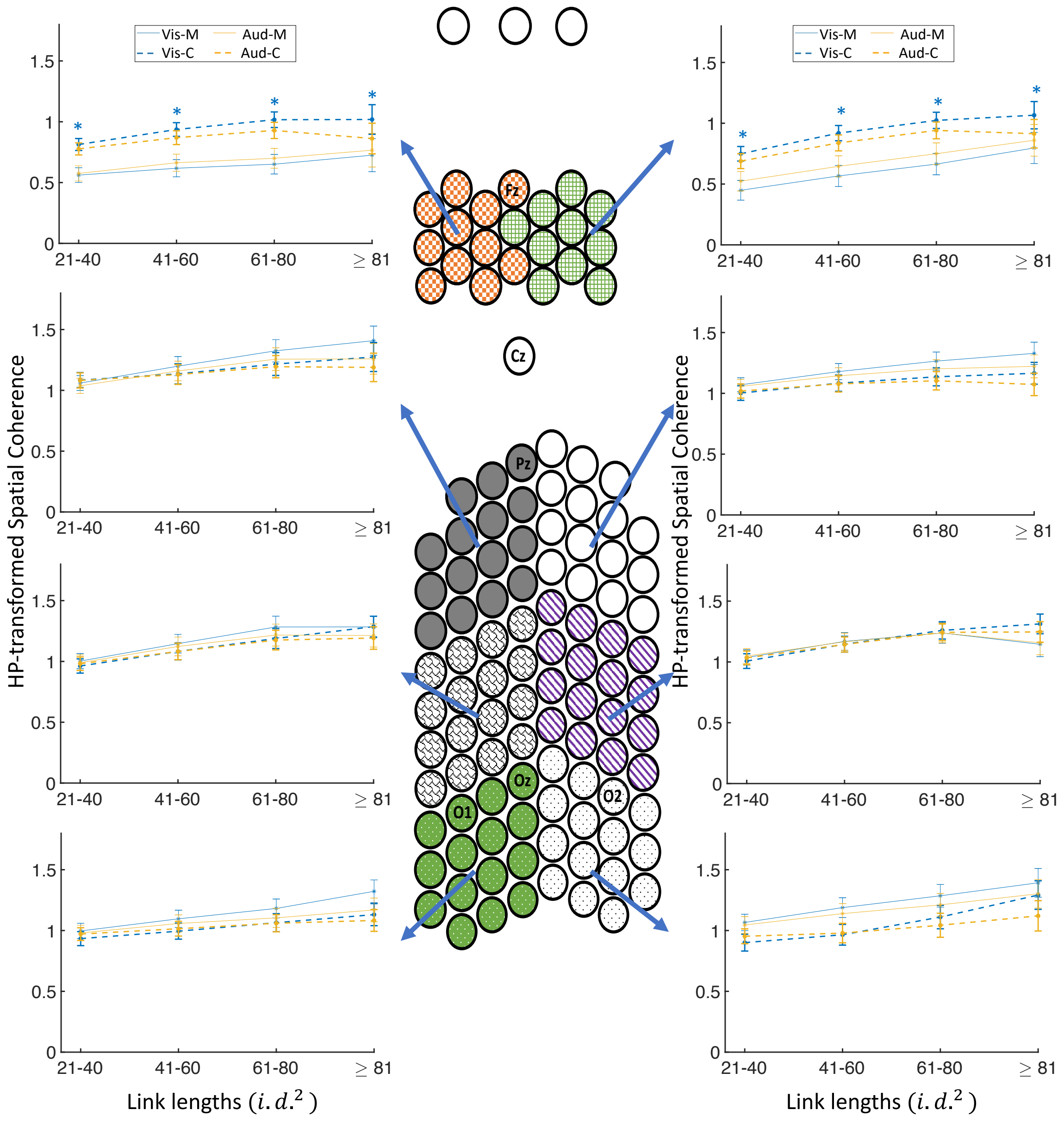

Supplementary Figure 15. Four-way normalized coherence interaction of group x spatial clusters x link lengths x modalities for 4Hz stimulation frequency. Comparison of spatial coherence between individuals with migraine and controls for the visual and auditory stimulation frequency of 4Hz as a function of link lengths for each of the eight spatial clusters, and groups. HP-transformed normalized spatial coherence is shown using dashed lines for controls and solid lines for the migraine patients. Asterisks show the significant group differences for each link length (on the x-axis) and each modality (colors of asterisks are matched with the modality), based on least significant difference (p<.04; LSD) post-hoc test (M= migraineurs, C=controls).

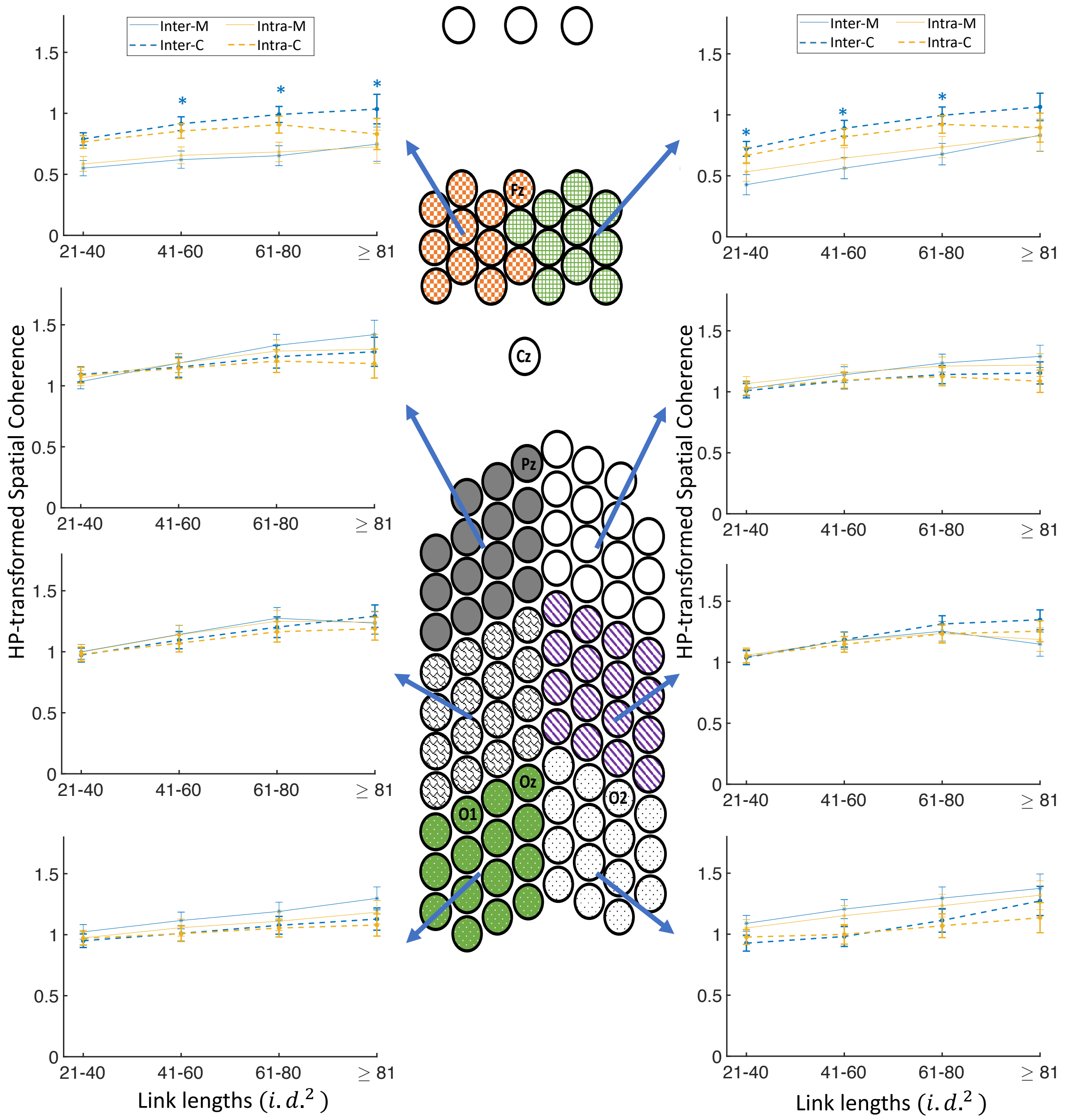

Supplementary Figure 16. Three-way normalized coherence interaction of group x spatial clusters x link lengths for 6Hz stimulation frequency. Comparison of spatial coherence between individuals with migraine and controls for the visual and auditory stimulation frequency of 6Hz as a function of link lengths for each of the eight spatial clusters, and groups. HP-transformed normalized spatial coherence is shown using dashed lines for controls and solid lines for the migraine patients. Asterisks show the significant group differences for each link length (on the x-axis) and each modality (colors of asterisks are matched with the modality), based on least significant difference ($p<.03$; LSD) post-hoc test (M= migraineurs, C=controls).

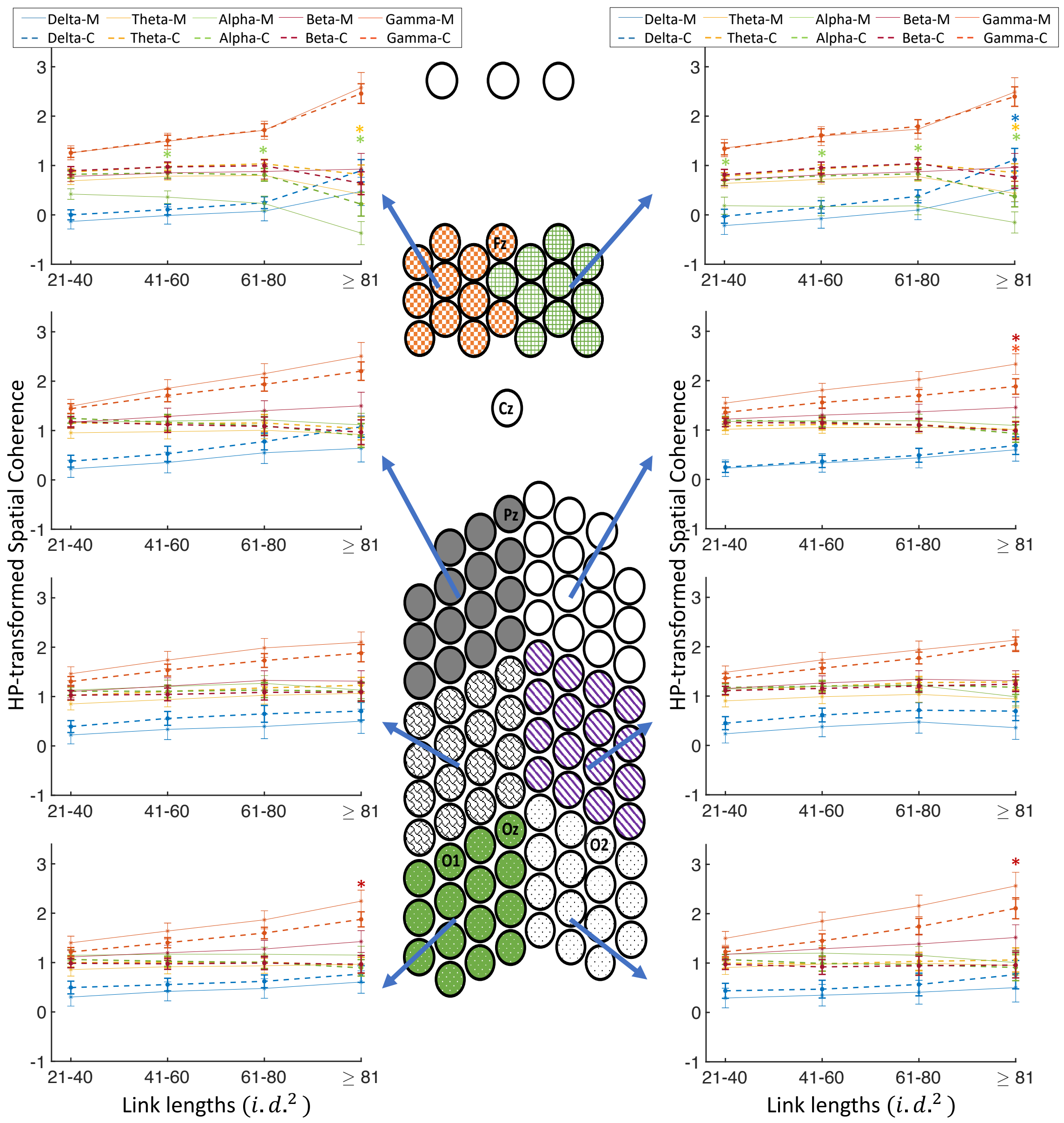

Supplementary Figure 17. Four-way resting-state normalized coherence interaction of group x frequency bands x spatial clusters x link lengths. Comparison of spatial coherence between individuals with migraine and controls during the resting-state recording as a function of link lengths for each of the five frequency bands, each of the eight spatial clusters, and groups (the four-way interaction of group x frequency bands x spatial clusters x link lengths). HP-transformed normalized spatial coherence is shown using dashed lines for controls and solid lines for the migraine patients. Asterisks show the significant group differences for each link length (on the x-axis) and each frequency band (colors of asterisks are matched with the frequency bands), based on least significant difference (p<.04; LSD) post-hoc test (M= migraineurs, C=controls).

Supplementary Note C. Power analysis:

To ensure that the sample size was sufficient for these analyses, we conducted a post-hoc power analysis for the mixed-model ANOVA tests. We used the open source *G*Power* software (Faul et al., 2007) to calculate the partial eta squared (PSE) of the significant interactions as defined by Cohen (1973), and the resultant power. The effect size (*f*) and power (*1-β*) of these interactions in the ANOVA test were estimated using *a=0.05*, number of groups=2 (migraineurs and controls), total sample size=28 (14 participants per group), and number of measurements=21 (total number of within factor levels in the visual/auditory coherence analysis; 19 for the resting-state recordings). Supplementary Table 1 summarizes the power analysis results.

Supplementary Table 1. *Estimation of effect size (f) and statistical power (1-β) for the significant interactions observed in the spatial coherence analysis of rest and sensory-evoked recordings (see Results, Coherence subsection for more details). G*Power software was used with a=0.05.*

Significant interaction	*f*	*1-β*
**4Hz stimulation frequency**		
group x hemisphere x cluster x link length x modality	0.25	0.99
group x hemisphere x frequency band x link length	0.27	0.99
group x hemisphere x frequency band x cluster	0.25	0.99
**6Hz stimulation frequency**		
group x cluster x link length	0.26	0.99
group x hemisphere x frequency band x link length	0.33	1.0
group x hemisphere x frequency band x cluster	0.26	0.99
**Resting-state**		
group x frequency band x cluster x link length	0.25	0.99

For the measures of coherence during visual, auditory, and resting-state recordings, the estimated statistical power of all of the significant interactions is ~1, which indicates that we have a sufficient sample size in our study.

Supplementary References:

Cohen, Jacob. "Eta-squared and partial eta-squared in fixed factor ANOVA designs." *Educational and psychological measurement* 33.1 (1973): 107-112.

Faul, Franz, et al. "G* Power 3: A flexible statistical power analysis program for the social, behavioral, and biomedical sciences." *Behavior research methods* 39.2 (2007): 175-191.
